# Identifying subgroups of frequent emergency department users: a latent class analysis with linked healthcare utilisation, cost and mortality outcomes in the UK

**DOI:** 10.1136/bmjph-2025-003920

**Published:** 2026-02-16

**Authors:** Richard Mattock, Chris Bojke, Samuel D Relton, Akshay Kumar, Chris Burton, Suzanne Mason, Sonia Saraiva, Robert West, William Lee, Christina van der Feltz-Cornelis, Catriona Marshall, Gerlinde Pilkington, Steven Ariss, Steven Dykes, Elspeth Guthrie

**Affiliations:** 1 Leeds Institute of Health Science, University of Leeds, Leeds, UK; 2 Lumanity, Sheffield, England, UK; 3 School of Medicine and Population Health, The University of Sheffield, Sheffield, UK; 4 Cornwall Partnership NHS Foundation Trust, Bodmin, UK; 5 Department of Health Sciences, University of York, York, UK; 6 University College London Institute of Health Informatics, London, UK; 7 Yorkshire Ambulance Service NHS Trust, Wakefield, UK

**Keywords:** Emergencies, Mental Health, Public Health, statistics and numerical data, Accidents

## Abstract

**Background:**

Frequent users (FUs) of emergency departments (EDs) attend repeatedly, placing a disproportionate burden on healthcare systems. Although known to be heterogeneous, there is limited international evidence characterising FU subpopulations or examining how healthcare costs and outcomes differ across groups. Advancing this understanding is important for developing tailored interventions to meet diverse care needs.

**Methods:**

FUs were defined as individuals with ≥5 ED attendances/year. We used two large UK datasets: Hospital Episode Statistics (HES, 2016–2019) and the Centre for Urgent and Emergency Care database (CUREd, 2017–2020). Together, these included over 148 000 FUs from 5 million ED users. Latent class analysis (LCA) was used to identify FU subgroups based on attendance patterns, healthcare use and diagnostic characteristics.

**Results:**

We identified three consistent subgroups (HES and CUREd): (1) low-severity FUs (n=23 034, 43.2%; n=7081, 32.7%); (2) high-intensity FUs with mental health and neurological needs (n=6288, 11.8%; n=3456, 15.9%); (3) older FUs with chronic illness and high inpatient use (n=24 028, 45.0%; n=11 139, 51.4%). Subgroups differed substantially in healthcare utilisation, costs and mortality. A fourth class varied across datasets: in HES, it showed moderate morbidity and complex needs; in CUREd, high morbidity and high-intensity ED use.

**Discussion:**

This is the first FU study to apply LCA across large-scale, multiyear ED datasets, identifying a potentially universal subgroup structure. Current services focus on a narrow subset of high-intensity users. Additional tailored strategies are needed to address the full spectrum of FU needs.

**WHAT IS ALREADY KNOWN ON THIS TOPIC**

- Frequent users (FUs) account for a disproportionately high number of emergency department (ED) attendances. They are a heterogeneous population who experience co-occurring health and social vulnerabilities.

**WHAT THIS STUDY ADDS**

- We use two large UK datasets and latent class analysis to characterise heterogeneity among FUs, identifying distinct subgroups with differing patterns of healthcare use, costs and mortality. We provide a potentially universal classification relevant across international settings.

**HOW THIS STUDY MIGHT AFFECT RESEARCH, PRACTICE OR POLICY**

- Identification of FU subgroups can inform tailored interventions, guide resource allocation and support policies to better integrate community and primary care with emergency services, ultimately reducing avoidable ED attendances and addressing population health inequalities.

## Background

 Rising demand for emergency department (ED) services is an increasing public health concern in many developed countries, placing pressure on resources, affecting the timeliness and quality of care, and prompting policies to shift appropriate care from acute to community settings.^1^ Within this context, frequent users (FUs) of EDs represent a small proportion of the overall patient population but account for a disproportionately high number of attendances.^2^ While there is no universally accepted definition of ‘frequent use’, studies often classify FUs as those attending three, four or five or more times per year.^3^

FUs represent a highly heterogeneous population with complex and often co-occurring health and social care needs.^4 5^ compared with non-FUs, they are more likely to experience a range of health and social vulnerabilities, including mental health conditions, substance use, multiple chronic illnesses, frailty and social disadvantage, which contribute to broader population health inequalities.^4 5^ Patterns of healthcare utilisation and associated costs also vary based on FU characteristics. For example, a UK-based study found that FUs with mental health conditions had higher ED attendances and greater use of emergency services, but were less likely to be admitted to hospital than those without mental health conditions.^6^

Studies^7^,^11^ from the USA, Canada and Italy have applied latent class analysis (LCA), a statistical technique that identifies subgroups within a population by analysing patterns across multiple observed variables,^12 13^ to formally examine heterogeneity among FUs. While some common subgroups emerged across studies, for example those with high utilisation and mental health problems,^7 8^ considerable variation also exists. Several unique subgroups were identified relating to specific physical diagnoses^7^; social vulnerabilities, such as housing instability or limited primary care access^11^; and demographics such as age.^10^ These differences likely reflect limitations in scope: three studies focused on highly specific patient subpopulations,^8 10 11^ one was confined to a single centre,^7^ and while one used a regional dataset, it included non-FUs in the analysis, resulting in a dominant latent class comprising 85% of the sample.^9^

Identifying consistent and reproducible FU subgroups in large population-wide datasets (eg, national) across multiple years is key to developing tailored interventions that are scalable and applicable across healthcare providers and diverse populations. Services for FUs tend to vary widely both across and within health systems. For example, in the UK, individual healthcare providers differ not only in the types of services they offer, such as hospital-based models, community care and the National Health Service (NHS) England High Intensity Use programme, but also in how they define and identify FUs.^14^ This inconsistency in intervention thresholds and service configuration reflects a lack of robust evidence. Notably, no formal statistical methods have yet been used to identify FU subgroups in the UK, leaving patient needs and treatment requirements unclear. A better understanding of this variability is key for designing efficient and equitable services that ensure no subgroups are marginalised or left without appropriate support.

The aims of this study are to conduct LCA in large UK population data to (1) identify subgroups of frequent ED users based on attendance patterns and patient characteristics; (2) describe these subgroups in terms of key demographic, clinical and social factors, along with patterns of healthcare utilisation, associated costs and mortality; and (3) demonstrate their consistency and reproducibility by comparing findings across two large datasets over multiple years.

## Methods

### Data sources and sample selection

This study draws on routinely collected healthcare data to support robust subgroup identification among frequent ED users. Two complementary datasets were used, each offering slightly different information relevant to identifying FU subgroups and assessing their associated outcomes.

The first source is Hospital Episode Statistics (HES), a national database of all NHS-funded patient care in England.^15^ ED attendances were identified from both the A&E Dataset and the Emergency Care Data Set, with duplicates across systems removed based on site, time and date. These records were linked to Admitted Patient Care, Outpatient, Medicines Dispensed in Primary Care and Office for National Statistics (ONS) mortality datasets, enabling a comprehensive overview of patient-level healthcare use, prescriptions and death registration.

The second source was the Centre for Urgent and Emergency Care database (CUREd), containing linked data from urgent and emergency care services across the Yorkshire and the Humber region, UK.^16^ It is structured similarly to HES and includes records of all ED attendances and inpatient admissions from acute NHS trusts, records from Yorkshire Ambulance Service of calls and attendances and finally NHS 111 telephone records.

CUREd data spanned April 2014 to March 2017. HES data covered April 2016 to March 2020. Annual ED attendance was defined by UK financial years (April–March), with the 2019/2020 HES data truncated at 29 February 2020 to exclude admissions occurring during the COVID-19 pandemic. Some variables, such as chronic frequent use, required data from the preceding year, meaning our analytical dataset covered 5 financial years: 2015/2016 (CUREd), 2016/2017 (CUREd), 2017/2018 (HES), 2018/2019 (HES), 2019/2020 (HES).

Because the national dataset was so large, a 10% random sample of HES ED patients was selected. All records from the regional CUREd database were used. The sample was limited to FUs who were defined as individuals with five or more ED attendances in a financial year. The threshold of five attendances was based on recommendations from a systematic review^3^ and our own (ongoing) realist review, which incorporated insights from published literature, patients and the public.

FUs were grouped by their main hospital trust, restricted to those with a Type-1 (ordinary) ED, resulting in the inclusion of 120 trusts in HES and 12 in CUREd. No exclusions were made based on patient characteristics. The final sample comprised approximately 50 000 FUs per year from HES and 20 000 per year from CUREd per year (online supplemental appendix 1). Since CUREd covers a regional subset of England, many individuals are expected to be included in both datasets.

### Indicator variables

Indicator variables were those used to identify subgroups of FUs in the LCA. These fell into three categories: ED attendance patterns, frequent use of other services and markers of patient health. Variables differed slightly between the HES and CUREd datasets due to differences in data availability.

#### ED attendance patterns

ED attendance patterns were captured using binary indicators available in both datasets. These included high-volume use (≥10 attendances), chronic use (≥5 attendances in both the current and prior year), multiprovider use (attending more than one ED in a year) and burst use (three or more attendances within any 30-day period).

An additional indicator for low-acuity ED attendances was included for the CUREd dataset only. This variable, previously derived within CUREd,^16^ identifies non-urgent or non-severe presentations. Individuals were classified as low-acuity users if any of their ED attendances during the year met this definition. To supplement this measure, we incorporated a binary ‘low inpatient rate’ variable which was available in both HES and CUREd datasets and defined as ‘yes’ when the ratio of inpatient admissions to ED attendances was less than 1:2 for a single individual.

#### Frequent use of other services

Binary indicators were used to capture frequent use of other healthcare services. For each variable, a threshold of five or more contacts per year was selected which classified approximately 25% as FUs. The indicators included frequent outpatient attendances (HES only), ambulance call-outs (CUREd only) and NHS 111 calls (CUREd only). Each was counted regardless of whether they resulted in/from ED attendances, to capture overall service use rather than only ED linked (or non-linked) events.

#### Health conditions

Health condition indicators were derived differently in HES and CUREd due to data availability. In HES, conditions were inferred from prescription records (available for 2018/2019 and 2019/2020 only), because ED diagnostic coding is often incomplete, sometimes with over 50% missing. Prescriptions were mapped at the British National Formulary (BNF) chapter level, with a condition flagged if any drug from a relevant chapter was dispensed during the year.^17^

Mental health conditions were identified based on the use of medications from BNF chapters 4.1 (hypnotics and anxiolytics), 4.2 (drugs used in psychoses and related disorders), 4.3 (antidepressants) and 4.7.1–4.7.2 (analgesics). Analgesics were included due to their potential use in managing psychosomatic symptoms, self-medication for psychological distress and substance misuse, particularly in the case of opioids (4.7.2). Drugs from other chapters with secondary psychiatric uses (eg, some antiepileptic drugs) were excluded to maintain a consistent and practical classification based on primary indication.

Cardiovascular conditions were captured using two groupings: (1) hypertension, heart failure or atrial fibrillation (BNF 2.1, 2.2, 2.5, 2.6.2, 2.8), and (2) coronary heart disease or other vascular disease (BNF 2.6.1, 2.6.3, 2.9). Respiratory conditions (asthma, chronic obstructive pulmonary disease) were identified from BNF 3.1, 3.2, 3.6; neurological conditions (eg, epilepsy, Parkinson’s) from BNF 4.8, 4.9; and diabetes from BNF 6.1.^17^

In CUREd, prescription data were unavailable, so health conditions were identified using a single physical comorbidities indicator derived from ED diagnostic codes, based on either the HES accident and emergency (A&E) or International Classification of Diseases, 10th Revision (ICD-10) coding schemes. Physical health conditions were classified using a summary variable indicating none, one or multiple morbidities, calculated by summing relevant physical diagnoses recorded within or across ED attendances. This approach is consistent with commonly used comorbidity indices such as the Charlson and Elixhauser measures.^18^ Full details of the diagnostic groupings are provided in online supplemental appendix 2.

### Postclassification measures

After identifying subgroups through LCA, we examined a range of post-classification measures to explore how these groups differed across key domains, including demographic characteristics, ED care characteristics, healthcare utilisation and costs, and mortality outcomes.

#### Demographic variables

Demographic measures were consistently defined across both HES and CUREd datasets. Age was recorded at individuals’ first ED attendance of the year and grouped into 10 bands: 0–14, 15–19, 20–24, 25–34, 35–44, 45–54, 55–64, 65–74, 75–84 and 85+ years old. Biological sex was recorded as male or female. Ethnicity was grouped into six categories: Asian, black, Mixed, Other, white and Unknown, following the classification in Pineda-Moncusí.^19^ Socioeconomic status was measured using the Index of Multiple Deprivation (IMD), a UK area-level composite measure based on income, employment, education, health, crime, housing and environment, reported in quintiles from most deprived (= 1) to least deprived (= 5).^20^

#### Emergency department care characteristics

A range of variables were derived to characterise patient health and the nature of emergency care received. For each measure, rather than simply recording whether FUs had ever received care in a given category, which could bias results due to varying numbers of attendances, we identified their most frequent (modal) category across all ED visits during the year.

ED diagnoses were recorded on three different coding systems (HES A&E codes, Systematized Nomenclature of Medicine Clinical Terms (SNOMED CT) and ICD-10) with variation both within and between the HES and CUREd datasets. To enable consistent analysis, we mapped all primary diagnoses to a unified set of broad attendance reason categories. This involved first mapping SNOMED codes to HES A&E codes and then condensing both HES A&E and ICD-10 codes to a common diagnostic framework. Full details of the mapping process are provided in online supplemental appendix 2. The resulting categories captured both chronic physical morbidities (aligned with comorbidity indicators described in online supplemental appendix A2.2) and other health-related presentations, including injury, infection, ear, nose and throat conditions, other miscellaneous diagnoses and cases where no abnormality was detected (online supplemental appendix A2.3). We also identified psychosocial conditions if any HES codes 35 (psychiatric) or 37 (social problems), or ICD-10 codes from chapter F or R45, were recorded during the year.

In addition, we examined the modal types of investigations and treatments received by FUs during their ED visits, arrival modes and discharge destination for each individual (eg, discharge home, hospital admission, referral elsewhere). Full category definitions and mappings across datasets for each of these variables are provided in online supplemental appendix 3.

#### Healthcare costs and utilisation

Data availability for healthcare costs differed between the HES and CUREd datasets. HES included costs for ED, inpatient, outpatient, critical care and primary care prescriptions, while CUREd covered ED, inpatient, ambulance and NHS 111 services. Utilisation was defined as the total number of recorded entries (admissions, appointments or prescriptions) per calendar year.

Unit costs were applied to each unit of resource use. For ED, inpatient, outpatient and critical care services, costs were derived from the NHS Improvement 2021/2022 National Cost Collection^21^ by linking healthcare resource groups to relevant currency codes in HES or CUREd. Ambulance call-outs (CUREd only) were categorised as ‘see and treat’ or ‘see and convey’ and costed using the corresponding unit cost categories.^21^ NHS 111 calls (CUREd only) were costed by multiplying total call duration by the hourly rate for Agenda for Change Band 5 staff, excluding qualification costs.^22^ Prescription costs were calculated by summing the provided ‘Actual Cost’ in the dataset of all items prescribed during the year, representing the amount paid by NHS commissioners after any reimbursement or negotiated pricing. Total annual costs were calculated by summing all available costed components and are reported for the year in which patients were identified as FUs.

#### Mortality

Mortality data were available for the HES dataset through linkage with the ONS mortality records. To explore survival trends, we plotted Kaplan-Meier survival curves by LCA subgroup, capturing 12-month survival from each individual’s fifth ED attendance (ie, the first instance they are classified as FUs) in the year.

### Data analysis

We conducted all data analysis in R V.4.2.0 using the poLCA package for LCA. Models were estimated using maximum likelihood estimation, with 20 random starts to avoid local minima and the solution with the lowest final log-likelihood selected as optimal. The poLCA package does not estimate bivariate residuals, therefore assuming local independence and no parameters were freed or constrained beyond the default. To determine the number of latent classes, we fitted models with progressively more classes and compared model fit using the Akaike information criterion (AIC) and Bayesian information criterion (BIC). The optimal number of classes was identified using the ‘elbow method’, where reductions in the AIC/BIC begin to plateau.^13^ To ensure robustness and interpretability of the model, we retained only solutions in which each class contained at least 5% of the total sample.^12^

A broad set of indicator variables was initially considered to capture the complexity of FU presentations, in line with best practices which support using a sufficient number of indicators to define meaningful subgroups.^12^ Several of these variables were then excluded due to high collinearity or limited contribution to model parsimony,^12 13^ such as mode of arrival, discharge destination and time of ED attendance.

Each individual was assigned to their most likely latent class based on the highest posterior probability. Following the class assignment, we calculated unadjusted descriptive statistics for each subgroup across various post-classification measures, including demographics, ED care characteristics, healthcare utilisation and costs, and mortality. Differences between subgroups were assessed using χ² tests for categorical variables and one-way analysis of variance for continuous variables.

The main analysis used the 2015/2016 year in CUREd and the 2018/2019 year in HES, as these correspond to the years for which we have complete data from both the previous and following years following FU identification, along with complete prescription data in HES (online supplemental appendix 1). A sensitivity analysis replicated the LCA for the 2016/2017 (CUREd) and 2019/2020 (HES) years. Missing data were minimal due to the nature of routinely collected datasets; therefore, imputation was not performed. The analysis was conducted and reported following best-practice recommendations for LCA (online supplemental appendix 4).^23^

## Results

### Model selection

Model fit improved with additional classes, as shown by decreasing AIC and BIC values. A clear flattening at the three-class solution indicated this as the ‘elbow point’, with only small improvements in fit observed beyond this in both the HES and CUREd datasets. While the four-class solution offered only marginal improvements in fit, it added interpretability by identifying a distinct additional subgroup. In contrast, the five-class model produced a small, unstable class (∼5% of the sample) with inconsistent membership across iterations.

**Figure 1:**
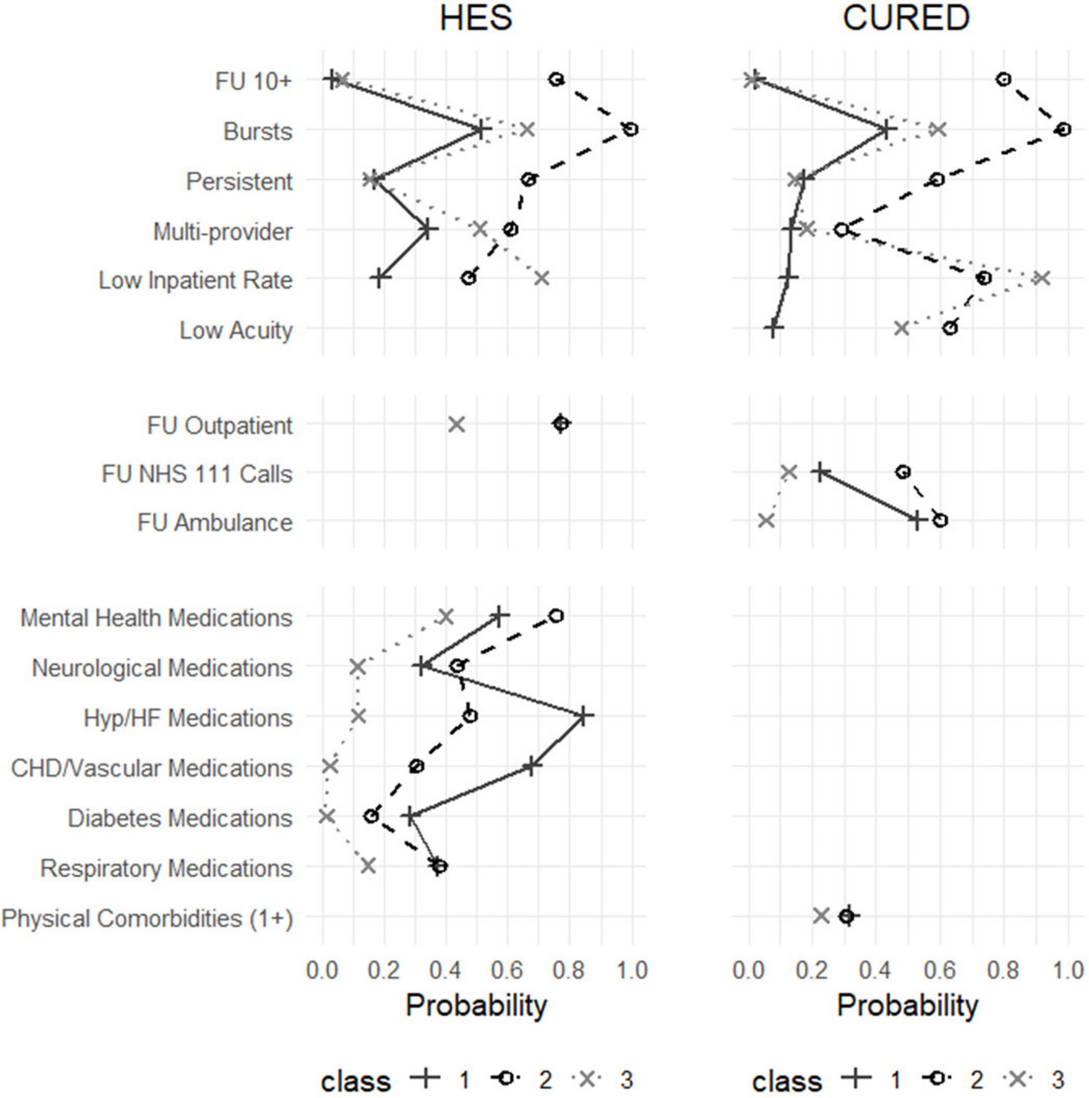
Latent class membership probabilities by indicator variables (three-class solution). Definitions: FU 10+ : ≥10 ED attendances per year; Bursts: ≥3 ED attendances per 30-days; Persistent: ≥5 ED attendances in current and previous year; Multiprovider: attended more than one ED per year; Low inpatient rate: <1:2 ratio of inpatient admissions to ED attendances; Low acuity: ≥1 non-urgent or non-severe ED admission per year; FU outpatient: ≥5 outpatient appointments per year; FU NHS 111 Calls: ≥5 NHS 111 calls per year; FU ambulance: ≥5 ambulance call-outs per year; Medications: any prescription for stated conditions (see online supplemental appendix 1); Physical comorbidities (1+): ≥1 distinct comorbidities recorded in ED diagnoses within a year (see online supplemental appendix 1). CHD, coronary heart disease; CUREd, Centre for Urgent and Emergency Care database; ED, emergency department; FU, frequent user; HES, Hospital Episode Statistics; HF, heart failure; Hyp, hypertension; NHS, National Health Service.

Based on these findings, results are presented for both the three-class and four-class models. Full AIC/BIC values are reported in online supplemental appendix 5. Class summaries, focusing on key patterns across indicator and outcome variables, are provided below. Class membership probabilities for indicator variables are shown in figures1  2 and detailed in online supplemental appendix 6. Postclassification differences in demographics, ED attendance, healthcare utilisation and costs are summarised in tables1  2 and further detailed in online supplemental appendices 7 and 8. Survival curves by class are shown in figure 3.

**Figure 2:**
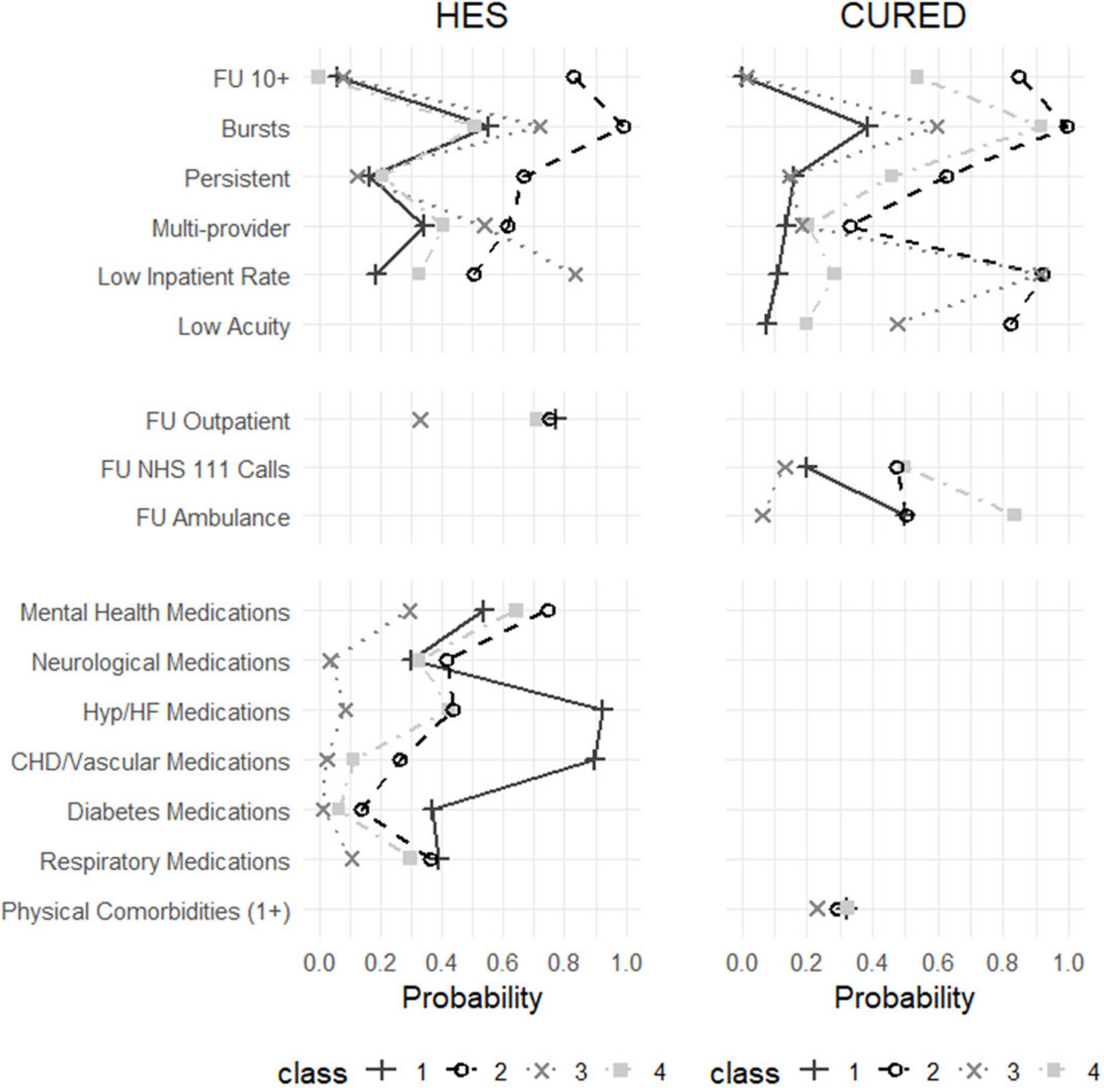
Latent class membership probabilities by indicator variables (four-class solution). Definitions: FU 10+ : ≥10 ED attendances per year; Bursts: ≥3 ED attendances per 30-days; Persistent: ≥5 ED attendances in current and previous year; Multiprovider: attended more than one ED per year; Low inpatient rate: <1:2 ratio of inpatient admissions to ED attendances; Low acuity: ≥1 non-urgent or non-severe ED admission per year; FU outpatient: ≥5 outpatient appointments per year; FU NHS 111 Calls: ≥5 NHS 111 calls per year; FU ambulance: ≥5 ambulance call-outs per year; Medications: any prescription for stated conditions (see online supplemental appendix 1); Physical comorbidities (1+): ≥1 distinct comorbidities recorded in ED diagnoses within a year (see online supplemental appendix 1). CHD, coronary heart disease; CUREd, Centre for Urgent and Emergency Care database; ED, emergency department; FU, frequent user; HES, Hospital Episode Statistics; HF, heart failure; Hyp, hypertension; NHS, National Health Service.

**Table 1:** Key characteristics by latent class membership, three-class solution; N (%)

Variable	HES (2018/2019)			CUREd (2015/2016)		
	Class 1, n=23 034, (43.2)	Class 2, n=6288, (11.8)	Class 3, n=24 028, (45.0)	Class 1, n=7081, (32.7)	Class 2, n=3456, (15.9)	Class 3, n=11 139, (51.4)
Demographic						
Age, mean (SD)	68.3 (18.3)	47.2 (19.8)	38.5 (17.6)	68.1 (20.1)	47.2 (20.7)	43.5 (20.8)
Female, n (%)	11 928 (52.0)	3434 (55.1)	12 605 (53.5)	3830 (54.1)	1680 (48.6)	5650 (50.7)
Lowest IMD*, n (%)	6801 (29.5)	2562 (40.7)	8919 (37.2)	3215 (45.4)	1979 (57.3)	5759 (51.8)
White ethnicity, n (%)	18 597 (80.7)	5116 (81.4)	16 949 (70.5)	6448 (91.1)	3091 (89.4)	9252 (83.1)
ED attendance reasons,† n (%)						
Central nervous system	481 (2.3)	225 (3.7)	500 (2.3)	101 (2.0)	52 (1.8)	122 (1.4)
Psychosocial	701 (3.3)	420 (7.0)	1131 (5.1)	76 (1.5)	114 (3.8)	259 (3.0)
Physical morbidities†	10 186 (48.0)	2071 (34.4)	6051 (27.2)	1246 (24.8)	423 (14.2)	1161 (13.2)
Non-physical‡	5122 (24.2)	1790 (29.8)	7905 (35.6)	792 (15.7)	383 (13.0)	2014 (22.9)
No classification/other§	4714 (22.2)	1517 (25.2)	6606 (29.8)	2835 (56.1)	1999 (67.3)	5217 (59.5)
ED ambulance arrival,† n (%)	13 423 (58.5)	3223 (51.4)	5349 (22.3)	5825 (82.3)	2275 (66.0)	3123 (28.3)
Discharged to home,† n (%)	10 097 (43.8)	4007 (63.7)	16 998 (70.7)	1058 (14.9)	1933 (55.9)	7939 (71.4)
Healthcare utilisation, mean (SD)						
ED	6.1 (1.5)	15.9 (13.5)	6.5 (3.7)	6.0 (1.2)	15.0 (10.9)	5.9 (1.2)
Inpatient¶	5.4 (9.1)	7.0 (9.3)	2.1 (3.7)	4.5 (1.7)	4.6 (4.2)	1.1 (1.2)
Outpatient	14.1 (15.8)	14.8 (17.4)	6.7 (9.8)	NA	NA	NA
Critical care	0.1 (0.5)	0.1 (0.6)	0.0 (0.2)	NA	NA	NA
Ambulance	NA	NA	NA	4.6 (3.2)	8.1 (9.9)	1.3 (1.8)
NHS 111 telephone	NA	NA	NA	3.1 (4.8)	10.1 (25.8)	2.1 (3.8)
Total costs, mean (SD)	£20 460 (£18 289)	£23 367 (£25 119)	£7679 (£11 404)	£18 287 (£11 382)	£17 075 (£14 826)	£4611 (£5646)

* The most deprived IMD quintile.

† Modal, that is, most frequent response recorded across all ED admissions. Significance markers not shown, all variables differed significantly by latent class (p<0.001).

† Summed total across cardiac, endocrine, gastrointestinal, genitourinary, respiratory and vascular/haematological categories (online supplemental appendix 2).

‡ Summed total across injury, infection, ear/nose/throat categories (online supplemental appendix 2).

§ Summed total across other, no classification and nothing abnormal categories (online supplemental appendix 2).

¶ HES includes all inpatient admissions, CUREd includes inpatient admissions linked to ED attendances.

CUREd, Centre for Urgent and Emergency Care database; ED, emergency department; HES, Hospital Episode Statistics; IMD, Index of Multiple Deprivation; NA, not assessed; NHS, National Health Service.

**Table 2:** Key characteristics by latent class membership, four-class solution; N (%)

Variable	HES (2018/2019)				CUREd (2016/2017)			
	Class 1, n=15 909, (29.8)	Class 2, n=5790, (10.9)	Class 3, n=16 078, (30.1)	Class 4, n=15 573, (29.2)	Class 1, n=6704, (30.9)	Class 2, n=2191, (10.1)	Class 3, n=11 381, (52.5)	Class 4, n=1400, (6.5)
Demographic								
Age, mean (SD)	71.8 (15.1)	45.8 (19.6)	36.0 (16.0)	51.9 (22.4)	68.2 (20.0)	41.4 (17.8)	43.6 (20.9)	60.9 (21.2)
Female, n (%)	7531 (47.6)	3124 (54.5)	7909 (50.4)	9403 (60.8)	3606 (53.8)	1038 (47.4)	5781 (50.8)	735 (52.5)
Lowest IMD*, n (%)	4757 (29.9)	2387 (41.2)	6149 (38.3)	4989 (32.1)	3044 (45.4)	1271 (58.1)	5888 (51.8)	750 (53.6)
White ethnicity, n (%)	12 650 (79.5)	4690 (81.0)	10 787 (67.1)	12 535 (80.5)	6108 (91.1)	1941 (88.6)	9461 (83.1)	1281 (91.5)
ED attendance reasons,† n (%)								
Central nervous system	272 (1.9)	188 (3.4)	195 (1.3)	551 (3.8)	95 (2.0)	33 (1.7)	125 (1.4)	22 (2.0)
Psychosocial	434 (3.0)	423 (7.6)	701 (4.7)	694 (4.8)	70 (1.5)	84 (4.3)	263 (2.9)	32 (2.9)
Physical morbidities†	7207 (49.3)	1830 (32.9)	3543 (23.9)	5728 (39.7)	1180 (24.9)	208 (10.5)	1191 (13.4)	251 (23.1)
Non-physical‡	3391 (23.2)	1702 (30.6)	5618 (37.9)	4106 (28.4)	756 (15.8)	278 (14.1)	2044 (22.8)	111 (10.2)
No classification/other§	3304 (22.6)	1414 (25.5)	4756 (32.1)	3363 (23.3)	2653 (55.7)	1369 (69.5)	5356 (59.7)	673 (61.8)
ED ambulance arrival,† n (%)	9590 (60.5)	2897 (50.1)	2517 (15.7)	6991 (45.1)	5475 (81.7)	1227 (56.2)	3264 (29.0)	1257 (89.8)
Discharged to home,† n (%)	6924 (43.5)	3799 (65.6)	12 112 (75.3)	8267 (53.1)	924 (13.8)	1562 (71.3)	8099 (71.3)	345 (24.7)
Healthcare utilisation, mean (SD)								
ED	6.3 (2.1)	16.8 (13.7)	6.6 (4.2)	6.0 (1.2)	6.0 (1.1)	16.9 (12.8)	6.0 (1.4)	11.1 (5.3)
Inpatient¶	5.6 (10.1)	6.9 (9.1)	1.3 (1.9)	4.5 (6.2)	4.5 (1.6)	3.3 (3.3)	1.1 (1.2)	6.8 (4.3)
Outpatient	14.2 (16.4)	14.2 (16.6)	4.6 (7.2)	12.6 (14.0)	NA	NA	NA	NA
Critical care	0.1 (0.5)	0.1 (0.6)	0.0 (0.1)	0.1 (0.4)	NA	NA	NA	NA
Ambulance	NA	NA	NA	NA	4.5 (3.1)	7.6 (11.3)	1.4 (1.9)	9.3 (6.1)
NHS 111 telephone	NA	NA	NA	NA	2.8 (4.5)	11.8 (30.1)	2.2 (5.5)	7.1 (9.8)
Total costs, mean (SD)	£21 097 (£18 782)	£23 030 (£25 525)	£4927 (£8678)	£16 297 (£16 046)	£18 368 (£11 347)	£13 277 (£11 753)	£4704 (£5736)	£24 352 (£16 348)

* The most deprived IMD quintile.

† Modal, that is, most frequent response recorded across all ED admissions. Significance markers not shown, all variables differed significantly by latent class (p<0.001).

† Summed total across cardiac, endocrine, gastrointestinal, genitourinary, respiratory and vascular/haematological categories (online supplemental appendix 2).

‡ Summed total across injury, infection, ear/nose/throat categories (online supplemental appendix 2).

§ Summed total across other, no classification and nothing abnormal categories (online supplemental appendix 2).

¶ HES includes all inpatient admissions, CUREd includes inpatient admissions linked to ED attendances.

CUREd, Centre for Urgent and Emergency Care database; ED, emergency department; HES, Hospital Episode Statistics; IMD, Index of Multiple Deprivation; NA, not assessed; NHS, National Health Service.

**Figure 3:**
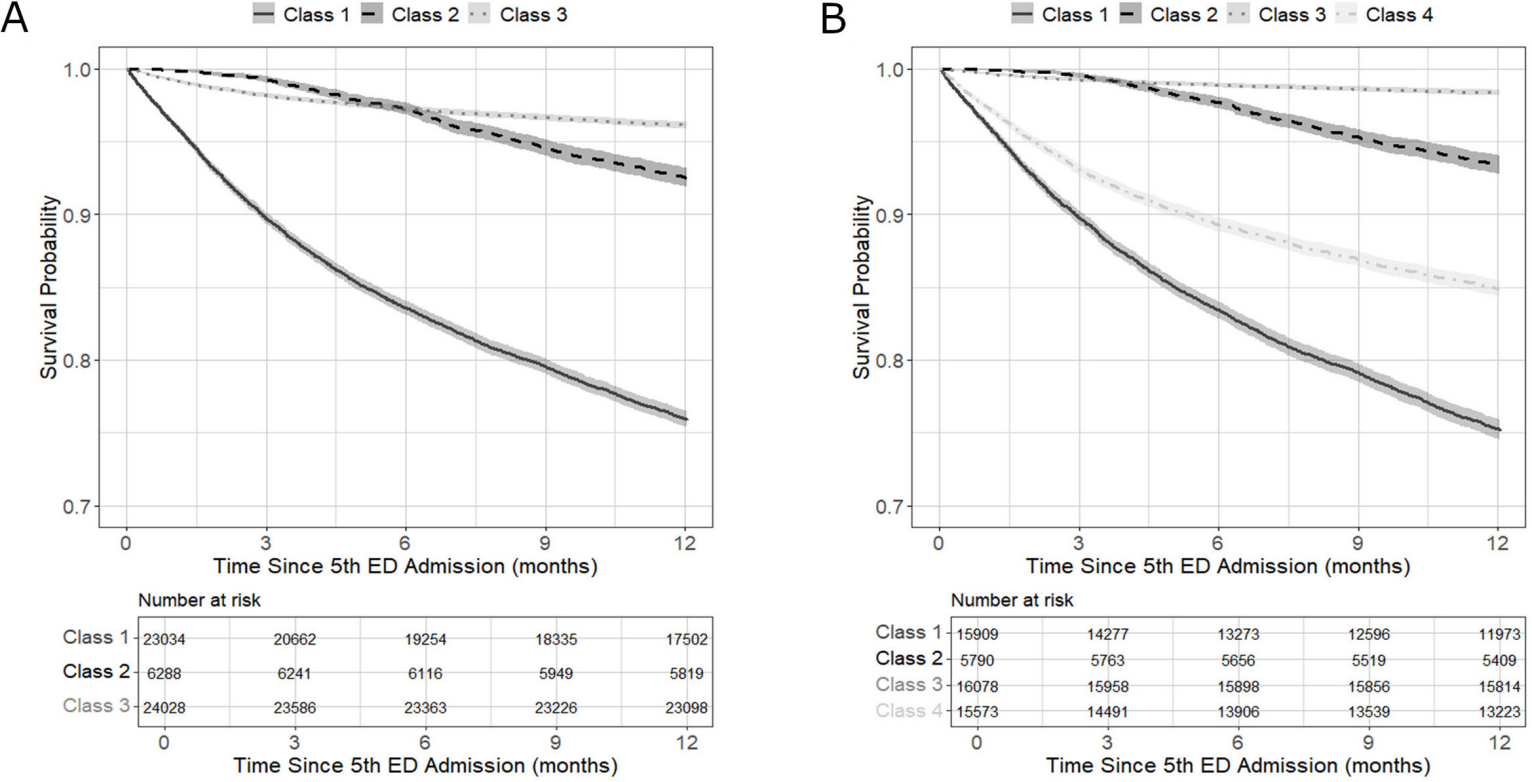
Survival curves by latent class. (A) Three-class solution; (B) Four-class solution. ED, emergency department.

### Three-class solution

The three-class solution identified distinct and interpretable groups with consistent profiles across HES and CUREd datasets.

#### Class 1: chronic, complex and frail FUs (HES: N=23 034, 43.2%; CUREd: N=7081, 32.7%)

This group had the highest inpatient admission rates and chronic comorbidity burden, particularly cardiovascular and diabetes-related conditions. Although not the most FUs, they showed high-severity ED use with extensive outpatient and ambulance service engagement. They were the least likely to be discharged home, were the oldest group (over one-third aged ≥80 years) and were least likely to be socioeconomically deprived. ED visits were mostly for medical rather than injury-related reasons, involving greater use of imaging and complex interventions than other classes. They had the highest overall costs, driven by inpatient admissions and the highest mortality (>28%), with steady decline from the fifth ED visit onward.

#### Class 2: high-intensity FUs (HES: N=6288, 11.8%; CUREd: N=3456, 15.9%)

These users were likely to be high volume FUs (≥10 visits), with persistent, burst and multiprovider use patterns. The group was mainly working-age adults, heavily concentrated in the most deprived IMD quintiles. Mental health and neurological medication use was prominent, as were ED visits for mental health concerns and low-acuity attendances. Investigation/treatment patterns were variable but often involved observation or pharmacological management. Service use was high across ED, NHS 111 and ambulance services, resulting in similarly high total costs as class 1. Mortality was 11%, with deaths occurring gradually after the fifth attendance.

#### Class 3: lower severity and morbidity FUs (HES: 24 028, N=45.0%; CUREd: 11 139, 51.4%)

This largest group had the lowest ED visit frequency, comorbidity and utilisation of other services. Attendances were often low-acuity, or injury-related, and typically involved fewer complex investigations. Treatment included an increased rate of minor surgical or musculoskeletal interventions. Less than 30% of FUs arrived by ambulance, and most were discharged home (>70%). They were the youngest class (35–40% under 30 years) and the most likely class to be from non-white ethnic backgrounds. Costs were the lowest overall, due to reduced inpatient use. Mortality was also lowest at 6%, though there was an early concentration of deaths shortly after the fifth ED visit, suggesting some acute, high-risk presentations.

### Four-class solution

In the four-class model, three classes (Classes 1, 2 and 3) remained broadly consistent across both datasets and closely mirrored those from the three-class solution. The introduction of a fourth class allowed greater differentiation among high-severity users, with differences in class characteristics across the datasets. In HES, Class 4A captured a moderate-morbidity group, incorporating individuals with lower acuity than the original Class 1 but greater needs than Class 3. In CUREd, Class 4B consisted largely of a reclassification of higher-acuity individuals from Class 2, forming a distinct group of high-intensity, high-need users.

#### Class 1: chronic, complex and frail FUs (HES: N=15 909, 29.8%; CUREd: N=6704, 30.9%)

This group retained similar characteristics to the same class in the three-class model including high costs driven by inpatient admissions and high mortality (29%).

#### Class 2: high-intensity FUs (HES: N=5790, 10.9%; CUREd: N=2191, 10.1%)

Still defined by frequent, persistent ED use and broad service engagement. Mortality in HES was 10%, with high costs across care settings. In CUREd, costs were lower due to reclassification (∼6%) of high-acuity users into Class 4B.

#### Class 3: lower severity and morbidity FUs (HES: N=16 078, 30.1%; CUREd: N=11 381, 52.5%)

In the HES data, many higher acuity users (∼20%) were reclassified into Class 4A, meaning this group had even lower mortality (2%) and reduced healthcare utilisation and costs. The CUREd group remained consistent with the three-class model.

#### Class 4A (HES): moderate morbidity, complex-need FUs (N=15 573, 29.2%)

Characterised by high inpatient and outpatient use, burst ED attendance and moderate overall severity. This group showed elevated use of mental health and neurological medications but relatively few medications for chronic physical comorbidities such as cardiovascular or diabetic conditions. The demographic was majority female (61%), White ethnicity, with relatively fewer from the most deprived areas. There were more ED attendances for central nervous system and gastrointestinal issues than other classes. Investigations and treatments were varied and complex. Overall service utilisation was moderate while mortality was the second highest (17%), likely reflecting the relatively old population (27%, ≥70 years).

#### Class 4B (CUREd): high-intensity, high-need FUs (N=1400, 6.5%)

Represented the smallest subgroup but had disproportionately high healthcare utilisation across ED, NHS 111 and ambulance services. Members were frequently high-volume, burst and persistent users. This older group (25% aged 80+) was predominantly white and over-represented in deprived areas, often presented with chronic physical conditions such as respiratory, cardiac and gastrointestinal issues, alongside a notable proportion with uncoded attendance reasons. They exhibited high multimorbidity and underwent extensive investigations and complex treatments, including respiratory and pharmacological management. This subgroup incurred the highest costs in the CUREd dataset, driven by both ED and inpatient admissions.

## Discussion

### Findings in context

This study applied LCA, a robust statistical method that models complex associations between variables,^12^ to identify distinct and reproducible subgroups of frequent ED users. In this context, LCA enabled the identification of subgroups defined by combinations of attendance characteristics, clinical profiles and medication usage, dimensions often examined separately in standard stratification methods. This approach provides a more detailed understanding of FU heterogeneity and could inform the development of targeted screening tools and tailored interventions to improve care pathways and optimise resource use.

To our knowledge, this study represents the largest, most comprehensive and most generalisable LCA of FUs to date. Using both national (HES) and large regional (CUREd) UK datasets, we analysed nearly 150 000 FUs across a 5-year period (2015/2016–2019/2020). Prior FU LCA studies from the USA, Canada and Italy have been limited by substantially smaller samples, shorter time frames and more restricted indicator variables.^7^,^11^ Their generalisability was also constrained by a focus on narrow patient subpopulations,^8 10 11^ single-centre settings (6) or broader samples that were dominated by non-FUs, which added noise to FU-specific pattern detection.^9^ In contrast, our study offers richer insights into patient profiles by examining diagnoses, care pathways (including treatments, investigations and discharge destinations), healthcare utilisation, costs and mortality risk.

Findings from our study, alongside evidence from other international settings, reveal a consistent and potentially universal pattern of FU subgroups (online supplemental appendix 9). Most notably, three subgroups recur: (1) a chronically ill, complex and often older group with high multimorbidity; (2) a high-intensity group with prevalent mental health or substance use needs; and (3) a younger and lower severity group with minimal morbidity and less persistent ED use. These three core classes consistently emerge across general population FU studies^7 8^ and within specific subpopulations, including geriatric^10^ and homeless^11^ FUs.

Beyond these core classes, additional heterogeneity reflects the population studied and available indicators. For example, distinct subgroups related to social vulnerabilities (ie, substance use, mental illness and trimorbidity) emerged among homeless FUs,^11^ while cancer-related groups appeared in geriatric populations.^10^ In our analysis, differences between the HES and CUREd datasets influenced the composition of the fourth class. The HES dataset included richer diagnostic data derived from medication records, while the CUREd dataset provided more detailed service utilisation measures such as ED acuity and use of telephone and ambulance services. Similarly, differences in indicator variable selection likely explain why Birmingham *et al* identified a cardiac-specific subgroup.^7^

### Policy implications

Identification of distinct LCA-derived subgroups enhances understanding of the mechanisms driving frequent ED use. Highlighting the differences between groups in terms of key characteristics like comorbidity profile, healthcare utilisation patterns and acuity can help inform the design of screening and interventions tailored to specific patient needs, improving outcomes for FUs.

Several of the attendance and diagnostic indicators in our analysis are readily observable by frontline staff, making them feasible for FU screening and triage. In the UK, high-intensity user programmes typically target individuals with ≥10 ED attendances per year, often combined with frequent 999 or NHS 111 use.^14 24^ This aligns with our high-intensity subgroup with complex mental health and neurological needs, which comprised only 10–15% of FUs. While many providers offer some form of liaison-based mental health and social support, provision is inconsistent across NHS trusts and frequently under-resourced.^14^

In contrast, older adults with frailty and chronic conditions who account for around 40% of all FUs and represent the largest costs and health burden often fall below current screening thresholds. Improved strategies are needed to identify these patients promptly, alongside the development of tailored interventions, such as integrated geriatric and chronic disease management, to address their complex needs. Evidence from health coaching^25^ and community-based care^26^ models suggests that coordinated multidisciplinary support can reduce hospitalisation and improve outcomes in frail elderly populations, but further research is needed to test these approaches specifically in FUs.

A further policy concern is whether frequent ED use can be reduced or redirected to more appropriate settings. Low-acuity (ie, the least severe and potentially avoidable) attendances were most common in the high-intensity class, recorded in 14% of all their ED attendances. Our LCA results found that these individuals had entrenched, repeated contact with both urgent and non-urgent services and likely high primary care use,^5^ suggesting a broader system-wide failure to meet complex needs. Targeted interventions for this group could include community care models that use integrated, holistic approaches extending beyond clinical care to include housing, mental health and social support.^27^ Without such coordinated approaches, persistent demand is unlikely to diminish.

The lower severity, lower morbidity group, comprising nearly half of all FUs, showed similarly high rates of low-acuity attendances and often presented as walk-ins and were discharged home. While most likely requiring some form of emergency care, these patterns suggest EDs sometimes function as substitutes for inaccessible or unsatisfactory primary care.^9^ Barriers such as long waits, digital exclusion and poor continuity of care disproportionately affect socioeconomically deprived and minority ethnic populations,^28^,^30^ who were over-represented in this group.

Tailored strategies to improve access to and engagement with primary and social care for these populations could help redirect demand, reduce pressure on EDs and address health inequalities. Redirecting all low-acuity attendances in the low severity, low morbidity group to more appropriate services would have substantial population impacts: scaling our 10% HES sample, this equates to approximately 200 000 fewer ED visits annually in England.

### Limitations

This study used two large datasets, enabling subgroup identification across multiple healthcare settings and outcomes. However, differences in available indicators (ie, NHS 111 and ambulance data in CUREd vs medication data in HES) introduced some variability: while the three-class solution produced consistent subgroups across both datasets, the fourth class differed in characteristics and offered limited explanatory value in the CUREd model. To support comparability across datasets and multiple coding schemes, we mapped ED features into broader categories; however, these were not clinically validated and may be open to different interpretations.

Deprivation, housing and other forms of social vulnerability are central drivers of frequent ED use.^11^ Although we showed that the most deprived individuals were disproportionately represented in Class 2 and Class 3 (online supplemental appendices 7.1 and 8.1), we did not comprehensively examine social impacts due to data availability in HES/CUREd. We attempted to directly incorporate IMD and other demographic factors using a latent class regression model, in which these variables predict class membership rather than define the classes; however, this model did not converge in our data. Future research is required to develop robust methods for integrating social vulnerability including housing instability, homelessness, social care involvement and addiction, into subgroup identification.

A further limitation is the lack of a universally accepted method for identifying mental health conditions from prescribing data, requiring trade-offs in classification. In our approach, including analgesics (especially non-opioids) may reduce specificity, while excluding other drug classes (eg, antiepileptics, attention deficit hyperactivity disorder medications) may reduce sensitivity. Nonetheless, minor changes to drug categorisation are unlikely to alter the overall findings, as post hoc analysis showed psychiatric ED diagnoses aligned well with latent classes based on mental health prescribing, supporting the classification’s validity.

To summarise ED attendances, we used the most frequent (ie, modal) category for each FU, though this approach reduced sensitivity to variation, as certain response codes were highly dominant. Missing data, particularly for ED diagnoses, further constrained analyses, though in HES, medication records were used to address this issue. Finally, although we included extensive data from urgent and emergency care, inpatient admissions, outpatient services and medication records, the absence of linked primary and community care data limited our ability to assess patterns of demand across the wider healthcare system.

### Conclusions

Our study identifies distinct and reproducible subgroups within frequent ED users and highlights their heterogeneity, complexity and differences in healthcare costs and mortality. Further understanding of these characteristics can help healthcare systems design targeted strategies to better manage demand and ease the growing pressure on EDs.

## Supplementary material

10.1136/bmjph-2025-003920 online supplemental file 1

## Data Availability

Data may be obtained from a third party and are not publicly available.
