# Supplementary material for: Identifying subgroups of frequent emergency department users: a latent class analysis with linked healthcare utilisation, cost and mortality outcomes in the UK
Source: BMJ Public Health. 2026 Feb 16;4(1):e003920. doi: 10.1136/bmjph-2025-003920 (PMC12911696; doi:10.1136/bmjph-2025-003920)
Supplement: online supplemental file 1 [file bmjph-4-1-s001.docx]

# Appendix 1:

**Table A1.1: Sample Selection and Data Availability**

|  | **HES** | | | | **CUREd** | | |
| --- | --- | --- | --- | --- | --- | --- | --- |
|  | **2016/17** | **2017/18** | **2018/19** | **2019/20** | **2014/15** | **2015/16** | **2016/7** |
| **Sample Selection** |  |  |  |  |  |  |  |
| ED Users^1^ | 1,349,851 | 1,392,594 | 1,768,125 | 1,842,003 | 884,542 | 895,952 | 900,502 |
| FUs | 48,793 | 53,567 | 65,361 | 59,940 | 22,492 | 23,531 | 24,201 |
| FUs of Acute NHS Trusts^2^ | 37,778 | 42,307 | 53,350 | 50,246 | 20,592 | 21,676 | 22,455 |
|  |  |  |  |  |  |  |  |
| **Data Availability** |  |  |  |  |  |  |  |
| Persistent FU status | No | Yes | Yes | Yes | No | Yes | Yes |
| Medication | No | No | Yes | Yes | No | No | No |
| ED acuity | No | No | No | No | Yes | Yes | Yes |
| 12-month mortality data | Yes | Yes | Yes | No | No | No | No |
|  |  |  |  |  |  |  |  |
| **Data analysis sample** | None | None | Main analysis | Sensitivity analysis | None | Main analysis | Sensitivity analysis |

*1: HES data represent a 10% random sample of all ED users between April 2016 and March 2020.*

*2: Records were excluded if an FU’s main provider (i.e. the most frequently attended trust) was not an Acute NHS Trust delivering ED care. The final HES sample included 120 Acute Trusts. For the CUREd dataset, one Acute Trust was excluded due to high levels of missing data, resulting in a total of 12 included Acute Trusts.*

# Appendix 2: Mapping ICD-10 & HES-AE Codes to Attendance Reason

**Table A2.1 Psychosocial Problems**

| **Attendance Reason** | **ICD-10 Code** | **ICD-10 Description** | **HES-A&E Code** | **HES-A&E Description** |
| --- | --- | --- | --- | --- |
| Psychosocial problems | Chapter F,  R45 | Mental and behavioural disorders | 35, 37 | Psychiatric problems,  social problems |

**Table A2.2 Physical Health Morbidities**

| **Attendance Reason** | **ICD-10 Code** | **ICD-10 Description** | **HES-A&E Code** | **HES-A&E Description** |
| --- | --- | --- | --- | --- |
| Cardiac Conditions | I30-I152 | Other forms of heart disease | 20 | Cardiac conditions |
| Central Nervous System conditions | Chapter G | Diseases of the nervous system | 24 | Central nervous system conditions (exc stroke) |
| Endocrine Conditions | Chapter E | Diabetes mellitus | 301, 302 | Diabetes, non-diabetic conditions |
| Gastrointestinal Conditions | Chapter K,  R10.0 | Diseases of the digestive system  Abdominal and pelvic pain | 26 | Gastrointestinal conditions |
| Genitourinary conditions | Chapter N | Symptoms and signs involving the urinary system | 27-29 | Urological, obstetric, gynaecological conditions |
| Respiratory Conditions | Chapter J | Diseases of the respiratory system | 25 | Respiratory conditions |
| Vascular/Haematological Conditions | Chapter I (excluding I30-I52) | Diseases of the circulatory system | 21-23 | Cerebrovascular, other vascular, haematological conditions |

The physical health comorbidities indicator variable was defined by mapping physical diagnoses to a broader attendance reason category. In total up to twelve diagnoses could be reported per ED admission. Patients were classified as having none, one, or multiple physical morbidities if they had multiple physical health attendance reasons recorded within the same ED admission, or multiple different attendance reasons across ED admissions within the year.

**Table A2.3 Non- Physical Health Morbidities**

| **Attendance Reason** | **ICD-10 Code** | **ICD-10 Description** | **HES-A&E Code** | **HES-A&E Description** |
| --- | --- | --- | --- | --- |
| Injury | Chapter S, Chapter T,  M79.9 | Injury, poisoning and certain other consequences of external causes  Soft tissue disorder, unspecified | 01-16 | Laceration  Contusion/abrasion  Soft tissue inflammation  Head injury  Dislocation/fracture/joint injury/amputation  Sprain/ligament injury  Muscle/tendon injury  Nerve injury  Vascular injury  Burns and scalds  Electric shock  Foreign body  Bites/stings  Poisoning (inc overdose)  Near drowning  Visceral injury |
| Infection/infectious disease | Chapter A,  Chapter B,  L08.9 | Certain infectious and parasitic diseases  Local infection of skin and subcutaneous tissue, unspecified | 17-19 | Infectious disease,  Local infection,  Septicaemia |
| Ear, Nose, Throat Conditions | Chapter H | Diseases of the eye and adnexa  Diseases of the ear and mastoid process | 34 | ENT conditions |
| Other conditions | All other codes left after above | General symptoms and signs | 31, 32, 33, 36 | Dermatological, allergy (including anaphylaxis), facio-maxillary conditions, ophthalmological conditions |
| Nothing abnormal detected | - | - | 39 |  |

# Appendix 3: Mapping ECDS (SNOMED) & HES-AE Codes

**Table A3.1 ED Investigation Codes**

| **Investigation Category** | **ECDS (SNOMED) description** | **ECDS (SNOMED) code** | **HES-AE (CDS-010) description** | **HES (CDS-010)**  **code** |
| --- | --- | --- | --- | --- |
| Administrative/ Unknown | Investigation not indicated | 1088291000000101 | None | 24 |
| Blood Gas Analysis | Venous blood gas | 61911006 | Arterial/capillary blood gas | 17 |
|  | Arterial / capillary blood gas | 60170009 |  |  |
|  | Lactate | 270982000 |  |  |
| Biochemistry | Urea & Electrolytes (U&Es) | 252167001 | Biochemistry | 05 |
|  | Bone profile | 167036008 |  |  |
|  | Liver function tests (LFTs) | 26958001 |  |  |
|  | Amylase | 89659001 |  |  |
|  | Lipase | 271232007 |  |  |
|  | Lipid profile | 16254007 |  |  |
|  | Glycosylated haemoglobin (HbA1c) | 43396009 |  |  |
|  | C-reactive protein (CRP) | 55235003 |  |  |
|  | Creatine kinase | 397798009 |  |  |
|  | Glucose | 104686004 |  |  |
|  | Thyroid function tests | 35650009 |  |  |
| Blood Typing | Cross match blood components | 252316009 | Cross match blood/group & save serum for later cross match | 04 |
| Cardiac Markers | Cardiac enzymes | 74500006 | Cardiac enzymes | 16 |
|  | Troponin | 105000003 |  |  |
| Cardiac Tests | Electrocardiogram | 29303009 | Electrocardiogram | 02 |
| Haematology | Full blood count (FBC) | 26604007 | Clotting studies | 14 |
|  | Thromboelastometry | 56027003 | Haematology | 03 |
|  | D-dimer | 70648006 |  |  |
|  | Erythrocyte sedimentation rate (ESR) | 416838001 |  |  |
|  | Clotting studies | 3116009 |  |  |
| Immunology | Immunology | 252375001 | Immunology | 15 |
|  | Mast cell tryptase | 62847008 | Serology | 20 |
|  | Serology | 68793005 |  |  |
| Microbiology | Bacteriology | 168338000 | Bacteriology | 07 |
|  | Blood culture | 30088009 | Blood culture | 19 |
|  | Swab for culture and sensitivities | 401294003 |  |  |
| Other (no mapping) | Peak expiratory flow | 29893006 | Histology | 08 |
|  | Dementia screening | 165320004 |  |  |
| Ophthalmology/Dental | Dental investigation | 53115007 | Dental investigation | 22 |
|  | Visual acuity testing | 16830007 | Refraction, orthoptic tests and computerised visual fields | 23 |
|  | Tonometry | 164729009 |  |  |
|  | Refraction, orthoptic and computerised visual fields | 86944008 |  |  |
|  | Intra-ocular fluid sampling | 363255004 |  |  |
|  | Ocular coherence tomography | 392010000 |  |  |
|  | Ocular photography | 282096008 |  |  |
| Other/ Unspecified | - | - | Other | 99 |
| Pregnancy Tests | Pregnancy test | 67900009 | Pregnancy test | 21 |
|  | Urine pregnancy test | 167252002 |  |  |
| Radiology/ Imaging | X-ray plain film | 168537006 | X-ray plain film | 1 |
|  | Ultrasound | 16310003 | Ultrasound | 10 |
|  | Computerised Tomography | 77477000 | Computerised tomography (exc genito urinary contrast examination/tomography) | 12 |
|  | Magnetic Resonance Imaging | 113091000 | Magnetic resonance imaging | 11 |
|  | Image intensifier investigation | 179929004 | Genito urinary contrast examination/tomography | 13 |
|  | Echocardiography | 40701008 |  |  |
| Toxicology | Toxicology | 269874008 | Toxicology | 18 |
| Urinalysis | Urinalysis | 27171005 | Urinalysis | 06 |

*Codes from individual schemes are grouped into broader investigation categories; however, they do not necessarily map directly between schemes. The codes shown reflect those observed in the dataset and may not include all possible investigation codes.*

**Table A3.2 ED Treatment Codes**

| **Investigation Category** | **ECDS (SNOMED) description** | **ECDS (SNOMED) code** | **HES-AE (CDS-010) description** | **HES (CDS-010)**  **code** |
| --- | --- | --- | --- | --- |
| Airway & respiratory support | Intubation: ETT or LMA | 112798008 | Intubation & Endotracheal tubes/laryngeal mask airways/rapid sequence induction | 15 |
|  | Nasal airway | 182692007 | Nasal airway | 38 |
|  | Supplemental oxygen | 57485005 | Oral airway | 39 |
|  | Non-invasive ventilation | 428311008 | Supplemental oxygen | 40 |
|  |  |  | CPAP/nasal intermittent positive pressure ventilation/bag valve mask | 41 |
| Critical cardiovascular care | Chest drain | 264957007 | Chest drain | 16 |
|  | Pleural aspiration | 91602002 | Defibrillation/pacing | 18 |
|  | Pleural drainage | 278296000 | Resuscitation/ cardiopulmonary resuscitation | 19 |
|  | External pacing | 18590009 |  |  |
|  | Defibrillation | 250980009 |  |  |
|  | Resuscitation / cardiopulmonary resuscitation | 439569004 |  |  |
|  | Percutaneous vascular occlusion (e.g.REBOA) | 240943006 |  |  |
|  | Resuscitative thoracotomy | 36936009 |  |  |
| Deprecated code | Code deprecated | Multiple deprecated codes | - | - |
| Decontamination/ poisoning management | Lavage / emesis / charcoal / eye irrigation | 226871000000103 | Lavage/ emesis/ charcoal/ eye irrigation | 14 |
|  | Eye : irrigation | 49999004 |  |  |
| Fracture and musculoskeletal care | Plaster of Paris: applied | 180289009 | Plaster of Paris | 05 |
|  | Plaster of Paris: removed | 180291001 | Splint | 06 |
|  | Application of splint | 79321009 | Physiotherapy | 09 |
|  | Physiotherapy: falls prevention | 391027005 | Manipulation | 10 |
|  | Physiotherapy: general | 430481008 | Fracture review | 33 |
|  | Manipulation upper limb fracture | 267765006 | Sling/collar cuff/broad arm sling | 36 |
|  | Manipulation lower limb fracture | 150617003 | Joint aspiration | 47 |
|  | Manipulation dislocation | 122944000 | Occupational therapy | 52 |
|  | Sling / collar cuff / broad arm sling | 52037006 | Loan of walking aid (crutches) | 53 |
|  | Joint aspiration | 90131007 |  |  |
|  | Occupational therapy: functional assessment | 304492001 |  |  |
|  | Occupational therapy: equipment provision / training | 410267000 |  |  |
|  | Provision of walking aid (e.g. crutches) | 243751002 |  |  |
| Guidance | Guidance / advice - written | 413334001 | Guidance/advice only | 22 |
| Invasive lines & fluid management | Intravenous cannula | 392231009 | Intravenous cannula | 12 |
|  | Intraosseous cannula | 430824005 | Central line | 13 |
|  | Central line | 233527006 | Urinary catheter/suprapubic | 17 |
|  | Urinary catheter / suprapubic | 410024004 | Arterial line | 42 |
|  | Gastrostomy tube change | 6125005 | Infusion fluids | 43 |
|  | Arterial line | 392247006 | Blood product transfusion | 44 |
|  | Infusion fluids | 103744005 | Pericardiocentesis | 45 |
|  | Blood product transfusion | 116859006 | Lumbar puncture | 46 |
|  | Lumbar puncture | 265232001 |  |  |
| Medication & pharmacological | Tetanus: toxoid - booster | 127786006 | Tetanus | 24 |
|  | Administration of human tetanus immune globulin | 572261000119106 | Nebulise/spacer | 25 |
|  | Nebuliser / spacer | 56251003 | Parenteral thrombolysis | 28 |
|  | Metered dose inhaler + spacer | 243132000 | Other parenteral drugs | 29 |
|  | Parenteral thrombolysis : tPA | 307521008 | Medication administered | 51 |
|  | Intravenous drug : bolus | 433215005 |  |  |
|  | Intravenous antibiotics | 281790008 |  |  |
|  | Intravenous drug : infusion | 432054008 |  |  |
|  | Administration of medication | 18629005 |  |  |
| Minor surgical interventions | Removal foreign body | 10849003 | Removal foreign body | 08 |
|  | Minor surgery | 711580002 | Incision and drainage | 11 |
|  | Eye: orthoptic exercises | 266740003 | Minor surgery | 20 |
|  | Eye: epilation of lashes | 74004007 | Eye | 55 |
|  | Eye: laser of retina / iris or posterior capsule | 35631009 | Dental treatment | 56 |
|  | Eye: subconjunctival injection | 74410004 | Prescription/medicines prepared to take away | 57 |
|  | Eye: retrobulbar injection | 121005 |  |  |
|  | Dental treatment | 81733005 |  |  |
|  | Prescription / medicines prepared to take away | 266712008 |  |  |
| None | Treatment not indicated | 183964008 | None (consider guidance/advice option) | 99 |
| Observation, monitoring, review | Cardiac monitor surveillance (regime/therapy) | 88140007 | Observation/electrocardiogram,pulse oximetry/head injury/trends | 21 |
|  |  |  | Recall/x-ray review | 32 |
| Other | Nasogastric tube | 87750000 | Other (consider alternatives) | 27 |
|  | Ascitic aspiration | 178016006 | Recording vital signs | 30 |
|  | Ascitic drainage | 178012008 |  |  |
|  | Psychosocial assessment | 371585000 |  |  |
|  | Medication review | 182836005 |  |  |
| Pain and sedation | Anaesthesia: general anaesthetic | 50697003 | Anaesthesia | 23 |
|  | Anaesthesia: local anaesthetic | 386761002 |  |  |
|  | Anaesthesia: entonox | 427035008 |  |  |
|  | Anaesthesia: regional block | 27372005 |  |  |
|  | Anaesthesia; sedation monitored | 398239001 |  |  |
| Social work | Social work intervention | 406551008 | Social worker intervention | 54 |
| Wound management | Dressing: wound / burn / eye | 15631002 | Dressing | 01 |
|  | Sutures: primary | 18557009 | Bandage/support | 02 |
|  | Sutures: complex / secondary | 71539001 | Sutures | 03 |
|  | Removal of sutures / clips | 30549001 | Wound closure (exc sutures) | 04 |
|  | Wound closure: steristrips | 71810007 | Burns review | 31 |
|  | Wound closure: glue | 284182000 | Wound cleaning | 34 |
|  | Wound closure: other e.g. staples | 50015006 | Dressing/wound review | 35 |
|  | Epistaxis control | 35807001 | Epistaxis control | 37 |
|  |  |  | Minor plastic procedure/splint skin graft | 48 |

*Codes from individual schemes are grouped into broader investigation categories; however, they do not necessarily map directly between schemes. The codes shown reflect those observed in the dataset and may not include all possible investigation codes.*

**Table A3.3 ED Arrival Mode Codes**

| **Arrival Mode Category** | **ECDS (SNOMED) description** | **ECDS (SNOMED) code** | **HES-AE (CDS-010) description** | **HES (CDS-010)**  **code** |
| --- | --- | --- | --- | --- |
| Ambulance (including helicopter) | Emergency road ambulance | 1048031000000100 | Brought in by Emergency Ambulance (including helicopter/'Air Ambulance') | 01 |
|  | Emergency road ambulance with medical escort | 1048041000000109 |  |  |
|  | Helicopter | 1048051000000107 |  |  |
| Other | Patient arranged own transport / walk-in (Arrival by own transport (finding)) | 1048071000000103 | Other | 02 |
|  | Public transport / taxi | 1048061000000105 |  |  |
|  | Non-emergency road ambulance | 1048021000000102 |  |  |
|  | Fixed wing / medical repatriation by air | 1048081000000101 |  |  |
|  | Custodial services : prison / detention centre transport | 1047991000000102 |  |  |
|  | Police transport | 1048001000000106 |  |  |

*ECDS (SNOMED) codes are mapped directly to CDS-010 as reported in “NHS Digital Mapping Definition to A&E (from ECDS)”*

**Table A3.4 ED Discharge Codes**

| **Discharge Category** | **ECDS (SNOMED) description** | **ECDS (SNOMED) code** | **HES-AE (CDS-010) description** | **HES (CDS-010)**  **code** |
| --- | --- | --- | --- | --- |
| Death | Admission to the mortuary (procedure) | 305398007 | Died in department | 10 |
| Discharged (home or elsewhere) | Discharge to home (procedure) | 306689006 | Discharged – follow-up treatment to be provided by general practitioner | 02 |
|  | Discharge to residential home (procedure) | 306691003 | Discharged – did not require any follow-up treatment | 03 |
|  | Discharge to nursing home (procedure) | 306694006 |  |  |
|  | Discharge to police custody (procedure) | 306705005 |  |  |
|  | Patient discharge, to legal custody (procedure) | 50861005 |  |  |
| Referred to ED ward | Emergency department discharge to emergency department short stay ward (procedure) | 1066331000000109 | Referred to A&E clinic | 04 |
| Referred ambulatory/ community care | Urgent admission to hospice (procedure) | 183919006 | Referred to other healthcare professional | 11 |
|  | Discharge to hospital at home service (procedure) | 1066351000000102 |  |  |
|  | Emergency department discharge to ambulatory emergency care service (procedure) | 1066341000000100 |  |  |
| Referred to inpatient ward | Discharge to ward (procedure) | 306706006 | Admitted to hospital bed / became a lodged patient of the same health care provider | 01 |
|  | Emergency department discharge to high dependency unit (procedure) | 1066361000000104 |  |  |
|  | Emergency department discharge to coronary care unit (procedure) | 1066371000000106 |  |  |
|  | Emergency department discharge to special care baby unit (procedure) | 1066381000000108 |  |  |
|  | Emergency department discharge to intensive care unit (procedure) | 1066391000000105 |  |  |
|  | Emergency department discharge to neonatal intensive care unit (procedure) | 1066401000000108 |  |  |
| Referred for procedure/outpatient | Emergency department discharge to operating theatre (procedure) | 1874161000000104 | Referred to fracture clinic | 05 |
|  |  |  | Referred to other outpatient clinic | 06 |
| Transferred | Patient transfer, to another health care facility (procedure) | 19712007 | Transferred to other healthcare provider | 07 |
| Other | Left department before being treated | 12 | Other | 14 |
|  | Left department having refused treatment | 13 |  |  |

*ECDS (SNOMED) codes are mapped directly to CDS-010 as reported in “NHS Digital Mapping Definition to A&E (from ECDS)”*

# Appendix 4: LCA Best Practice Reporting Checklist

**Table A4.1**

| **Item** | **Description** | **Location in text** |
| --- | --- | --- |
| #1 | Show Evaluative Information for all models tested  1a) Covariance Matrix  1b) Profiles  1c) Percentage in each class  1d) Evaluative criteria  1e) Residual analysis | 1a) Appendix A5.4 & A5.5  1b) Figures 1 & 2  1c) Class descriptions (results section 3.2 & 3.3)  1d) Methods (section 2.4)  1e) N/A – explanation provided in Methods (section 2.4) |
| #2 | Explain reasoning choices for choosing a specific model | Methods (section 2.4), Results (section 3.1), Appendix 5. |
| #3 | Explain choices related to “fixing” a bivariate residual relationship to zero | Methods (section 2.4) |
| #4 | Provide software used and the version number | Methods (section 2.4) |
| #5 | Submit the traditional descriptive and frequency data | Appendix 4 (A5.3) descriptives for indicator variables, Appendix 7-8 descriptives for outcome variables. |
| #6 | Include all relevant information and additional analyses in manuscript | Full results for 3-class and 4-class solution across multiple years reported in Results (Fig 1-3), Table 1&2, Appendix 6-8 |
| #7 | Include comprehensive literature and theoretical references | Reference list, Appendix 9. |

Reporting guidelines for LCA from Schreiber et al. (2017)(23).

# Appendix 5: Descriptives/ Diagnostics

**Figure A5.1 Bayesian Information Criterion (BIC) Plot by Class Number HES**

*
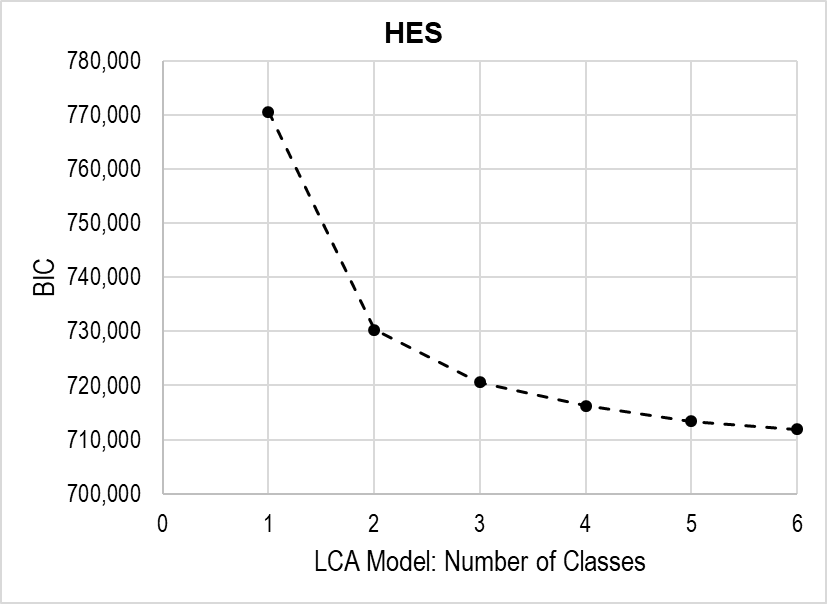
*

**Figure A5.2 Bayesian Information Criterion (BIC) Plot by Class Number HES**


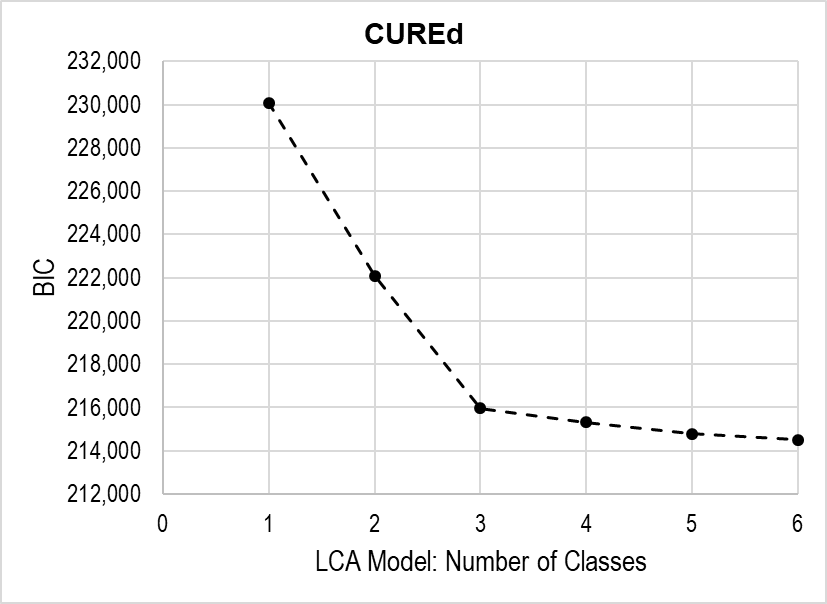


**Table A5.3: Descriptive Summary: Indicator Variables**

| **Variable** | **HES (2018/19)**  **N = 53,350** | **CUREd**  **(2016/17)**  **N = 21,676** |
| --- | --- | --- |
| Low Inpatient Rate  No  Yes | 29,130 (55%)  24,220 (45%) | 8,099 (37%)  13,577 (63%) |
| Burst Usage  No  Yes | 19,115 (36%)  34,235 (64%) | 8,602 (40%)  13,074 (60%) |
| Multi-provider Usage  No  Yes | 29,329 (55%)  24,021 (45%) | 17,659 (81%)  4,017 (19%) |
| Persistent Usage  No  Yes | 41,388 (78%)  11,962 (22%) | 16,772 (77%)  4,904 (23%) |
| FU 10+  No  Yes | 45,917 (86%)  7,433 (14%) | 18,614 (86%)  3,062 (14%) |
| Mental Health Medications  No  Yes | 25,614 (48%)  27,736 (52%) | N/A  N/A |
| Neurological Medications  No  Yes | 40,337 (76%)  13,013 (24%) | N/A  N/A |
| Diabetes Medications  No  Yes | 45,402 (85%)  7,948 (15%) | N/A  N/A |
| Hyp/ HF Medications  No  Yes | 27,975 (52%)  25,375 (48%) | N/A  N/A |
| CHD/ Vascular Medications  No  Yes | 35,177 (66%)  18,173 (34%) | N/A  N/A |
| Respiratory Medications  No  Yes | 38,617 (72%)  14,733 (28%) | N/A  N/A |
| Low Acuity  No  Yes | N/A  N/A | 13,642 (63%)  8,034 (37%) |
| FU Outpatient  No  Yes | 20,230 (38%)  33,120 (62%) | N/A  N/A |
| FU NHS 111 Calls  No  Yes | N/A  N/A | 16,985 (78%)  4,691 (22%) |
| FU Ambulance  No  Yes | N/A  N/A | 15,163 (70%)  6,513 (30%) |
| Physical Comorbidities (1+)  No  Yes | N/A  N/A | 15,832 (73%)  5,844 (27%) |

**Table A5.4: Covariance Matrix – HES 2018/19**

| **Variable** | Low  Inpatient  Rate | FU  Outpatient | Burst  Usage | Multi-provider  Usage | Persistent  Usage | FU 10+ | Mental Health  Meds. | Neuro. Meds. | Dia. Meds. | Hyp/  HF  Meds. | CHD/ Vasc Meds. | Resp.  Meds. |
| --- | --- | --- | --- | --- | --- | --- | --- | --- | --- | --- | --- | --- |
| Low Inpatient Rate | 0.25 |  |  |  |  |  |  |  |  |  |  |  |
| FU Outpatient | -0.07 | 0.24 |  |  |  |  |  |  |  |  |  |  |
| Burst Usage | 0.03 | -0.01 | 0.23 |  |  |  |  |  |  |  |  |  |
| Multi-provider Usage | 0.03 | 0.00 | 0.02 | 0.25 |  |  |  |  |  |  |  |  |
| Persistent Usage | 0.00 | 0.01 | 0.17 | 0.01 | 0.25 |  |  |  |  |  |  |  |
| FU 10+ | 0.01 | 0.01 | 0.05 | 0.02 | 0.12 | 0.25 |  |  |  |  |  |  |
| Mental Health Medications | -0.02 | -0.02 | 0.00 | 0.00 | 0.02 | 0.02 | 0.25 |  |  |  |  |  |
| Neurological Medications | -0.03 | 0.03 | -0.01 | -0.01 | 0.01 | 0.01 | 0.01 | 0.25 |  |  |  |  |
| Diabetes Medications | -0.03 | 0.03 | -0.01 | -0.01 | 0.01 | 0.00 | 0.01 | 0.01 | 0.25 |  |  |  |
| Hyp/ HF Medications | -0.09 | 0.05 | -0.02 | -0.03 | 0.00 | 0.00 | 0.03 | 0.03 | 0.05 | 0.25 |  |  |
| CHD/ Vascular Medications | -0.07 | 0.05 | -0.02 | -0.02 | 0.00 | 0.00 | 0.02 | 0.02 | 0.06 | 0.12 | 0.22 |  |
| Respiratory Medications | -0.03 | -0.03 | -0.01 | -0.01 | 0.02 | 0.01 | 0.02 | 0.02 | 0.03 | 0.04 | 0.03 | 0.20 |

*Hyp = Hypertension; HF = Heart Failure, CHD = Coronary Heart Disease*

**Table A5.5: Covariance Matrix – CUREd 2016/17**

| **Variable** | Low Inpatient Rate | Burst Usage | Multi-provider Usage | Persistent Usage | FU 10+ | Low Acuity | FU NHS 111 Calls | FU Ambulance | Physical Comorb. (1+) |
| --- | --- | --- | --- | --- | --- | --- | --- | --- | --- |
| Low Inpatient Rate | 0.23 |  |  |  |  |  |  |  |  |
| Burst Usage | 0.04 | 0.24 |  |  |  |  |  |  |  |
| Multi-provider Usage | 0.01 | 0.01 | 0.15 |  |  |  |  |  |  |
| Persistent Usage | 0.00 | 0.02 | 0.01 | 0.18 |  |  |  |  |  |
| FU 10+ | 0.01 | 0.05 | 0.01 | 0.05 | 0.12 |  |  |  |  |
| Low Acuity | 0.08 | 0.03 | 0.01 | 0.02 | 0.03 | 0.23 |  |  |  |
| FU NHS 111 Calls | 0.00 | 0.01 | 0.01 | 0.03 | 0.03 | 0.03 | 0.17 |  |  |
| FU Ambulance | -0.08 | 0.01 | 0.00 | 0.03 | 0.04 | 0.04 | 0.05 | 0.21 |  |
| Physical Comorbidities (1+) | -0.03 | 0.00 | 0.00 | 0.01 | 0.01 | 0.01 | 0.02 | 0.00 | 0.42 |

# Appendix 6: Latent Class Membership Probabilities by Indicator Variables

**Table A6.1: HES, three-class solution**

|  | **HES (2018/19)** | | | **HES (2019/20) *** | | |
| --- | --- | --- | --- | --- | --- | --- |
| **Indicator Variable** | **Class 1,**  **N = 23,034,**  **(43.2%)** | **Class 2,**  **N =6,288,**  **(11.8%)** | **Class 3,**  **N = 24,028,**  **(45.0%)** | **Class 1,**  **N = 22,651**  **(45.1%)** | **Class 2,**  **N =5,737**  **(11.4%)** | **Class 3,**  **N = 21,858**  **(43.5%)** |
| FU 10+ | 0.03 | 0.76 | 0.06 | 0.04 | 0.73 | 0.06 |
| Burst Usage | 0.52 | 1.00 | 0.66 | 0.55 | 1.00 | 0.69 |
| Persistent Usage | 0.17 | 0.66 | 0.15 | 0.19 | 0.73 | 0.17 |
| Multi-provider Usage | 0.34 | 0.61 | 0.51 | 0.35 | 0.61 | 0.50 |
| Low Inpatient Rate | 0.19 | 0.47 | 0.71 | 0.20 | 0.53 | 0.71 |
| Low Acuity | NA | NA | NA | NA | NA | NA |
| FU Outpatient | 0.77 | 0.77 | 0.43 | 0.75 | 0.70 | 0.41 |
| FU NHS 111 Calls | NA | NA | NA | NA | NA | NA |
| FU Ambulance | NA | NA | NA | NA | NA | NA |
| Mental Health Medications | 0.57 | 0.76 | 0.40 | 0.57 | 0.78 | 0.41 |
| Neurological Medications | 0.32 | 0.43 | 0.12 | 0.32 | 0.42 | 0.12 |
| Hyp/ HF Medications | 0.84 | 0.48 | 0.12 | 0.84 | 0.45 | 0.13 |
| CHD/ Vascular Medications | 0.68 | 0.31 | 0.02 | 0.68 | 0.26 | 0.03 |
| Diabetes Medications | 0.28 | 0.16 | 0.01 | 0.29 | 0.14 | 0.02 |
| Respiratory Medications | 0.38 | 0.38 | 0.15 | 0.38 | 0.39 | 0.15 |
| Physical Comorbidities (1+) | NA | NA | NA | NA | NA | NA |

*Table reports class-conditional response probabilities for each indicator variable. Only probabilities of a “Yes” response are shown (i.e., P[Yes] given class membership).*

*Hyp = Hypertension; HF = Heart Failure, CHD = Coronary Heart Disease*

**Data truncated and covered 11-months from 1^st^ April 2019 to 29^th^ February 2020 due to COVID-19 pandemic.*

**Table A6.2: CURED, three-class solution**

|  | **CUREd (2015/16)** | | | **CUREd (2016/17)** | | |
| --- | --- | --- | --- | --- | --- | --- |
| **Indicator Variable** | **Class 1,**  **N = 7,081,**  **(32.7%)** | **Class 2,**  **N =3,456,**  **(15.9%)** | **Class 3,**  **N = 11,139,**  **(51.4%)** | **Class 1,**  **N = 7,737**  **(34.5%)** | **Class 2,**  **N = 3,368**  **(15.0%)** | **Class 3,**  **N = 11,350**  **(50.1%)** |
| FU 10+ | 0.02 | 0.80 | 0.01 | 0.03 | 0.79 | 0.02 |
| Burst Usage | 0.43 | 0.99 | 0.59 | 0.44 | 0.99 | 0.59 |
| Persistent Usage | 0.18 | 0.59 | 0.14 | 0.18 | 0.57 | 0.16 |
| Multi-provider Usage | 0.14 | 0.29 | 0.18 | 0.13 | 0.30 | 0.19 |
| Low Inpatient Rate | 0.13 | 0.74 | 0.92 | 0.12 | 0.76 | 0.92 |
| Low Acuity | 0.08 | 0.63 | 0.48 | 0.08 | 0.67 | 0.49 |
| FU Outpatient | NA | NA | NA | NA | NA | NA |
| FU NHS 111 Calls | 0.22 | 0.49 | 0.13 | 0.24 | 0.49 | 0.14 |
| FU Ambulance | 0.53 | 0.60 | 0.06 | 0.56 | 0.61 | 0.07 |
| Mental Health Medications | NA | NA | NA | NA | NA | NA |
| Neurological Medications | NA | NA | NA | NA | NA | NA |
| Hyp/ HF Medications | NA | NA | NA | NA | NA | NA |
| CHD/ Vascular Medications | NA | NA | NA | NA | NA | NA |
| Diabetes Medications | NA | NA | NA | NA | NA | NA |
| Respiratory Medications | NA | NA | NA | NA | NA | NA |
| Physical Comorbidities (1+) | 0.32 | 0.31 | 0.23 | 0.33 | 0.31 | 0.24 |

*Table reports class-conditional response probabilities for each indicator variable. Only probabilities of a “Yes” response are shown (i.e., P[Yes] given class membership).*

*Hyp = Hypertension; HF = Heart Failure, CHD = Coronary Heart Disease*

**Table A6.3: HES, four-class solution**

|  | **HES (2018/19)** | | | | **HES (2019/20)*** | | | |
| --- | --- | --- | --- | --- | --- | --- | --- | --- |
| **Indicator Variable** | **Class 1,**  **N = 15,909,**  **(29.8%)** | **Class 2,**  **N =5,790,**  **(10.9%)** | **Class 3,**  **N = 16,078,**  **(30.1%)** | **Class 4,**  **N = 15,573,**  **(29.2%)** | **Class 1,**  **N =15,897,**  **(31.6%)** | **Class 2,**  **N =5,220,**  **(10.4%)** | **Class 3,**  **N = 14,767,**  **(29.4%)** | **Class 4,**  **N = 14,362,**  **(28.6%)** |
| FU 10+ | 0.06 | 0.83 | 0.08 | 0.00 | 0.06 | 0.78 | 0.08 | 0.00 |
| Burst Usage | 0.55 | 0.99 | 0.72 | 0.51 | 0.59 | 1.00 | 0.75 | 0.53 |
| Persistent Usage | 0.17 | 0.66 | 0.12 | 0.21 | 0.19 | 0.71 | 0.15 | 0.22 |
| Multi-provider Usage | 0.34 | 0.61 | 0.54 | 0.41 | 0.35 | 0.62 | 0.53 | 0.39 |
| Low Inpatient Rate | 0.18 | 0.50 | 0.83 | 0.33 | 0.20 | 0.55 | 0.83 | 0.32 |
| Low Acuity | NA | NA | NA | NA | NA | NA | NA | NA |
| FU Outpatient | 0.77 | 0.75 | 0.33 | 0.71 | 0.75 | 0.68 | 0.32 | 0.69 |
| FU NHS 111 Calls | NA | NA | NA | NA | NA | NA | NA | NA |
| FU Ambulance | NA | NA | NA | NA | NA | NA | NA | NA |
| Mental Health Medications | 0.54 | 0.74 | 0.30 | 0.64 | 0.53 | 0.77 | 0.31 | 0.64 |
| Neurological Medications | 0.30 | 0.41 | 0.04 | 0.33 | 0.30 | 0.40 | 0.04 | 0.33 |
| Hyp/ HF Medications | 0.92 | 0.44 | 0.09 | 0.42 | 0.91 | 0.42 | 0.10 | 0.44 |
| CHD/ Vascular Medications | 0.90 | 0.26 | 0.02 | 0.11 | 0.90 | 0.23 | 0.03 | 0.12 |
| Diabetes Medications | 0.37 | 0.14 | 0.01 | 0.06 | 0.37 | 0.12 | 0.02 | 0.06 |
| Respiratory Medications | 0.39 | 0.36 | 0.11 | 0.30 | 0.39 | 0.37 | 0.11 | 0.31 |
| Physical Comorbidities (1+) | NA | NA | NA | NA | NA | NA | NA | NA |

*Table reports class-conditional response probabilities for each indicator variable. Only probabilities of a “Yes” response are shown (i.e., P[Yes] given class membership).*

*Hyp = Hypertension; HF = Heart Failure, CHD = Coronary Heart Disease.*

**Data truncated and covered 11-months from 1^st^ April 2019 to 29^th^ February 2020 due to COVID-19 pandemic.*

**Table A6.4: CUREd, four-class solution**

|  | **CUREd (2015/16)** | | | | **CUREd (2016/17)** | | | |
| --- | --- | --- | --- | --- | --- | --- | --- | --- |
| **Indicator Variable** | **Class 1,**  **N = 6,704,**  **(30.9%)** | **Class 2,**  **N =2,191,**  **(10.1%)** | **Class 3,**  **N = 11,381,**  **(52.5%)** | **Class 4,**  **N = 1,400,**  **(6.5%)** | **Class 1,**  **N = 5,688,**  **(25.3%)** | **Class 2,**  **N =2,922,**  **(13.0%)** | **Class 3,**  **N = 11,759,**  **(52.4%)** | **Class 4,**  **N = 2,086,**  **(9.3%)** |
| FU 10+ | 0.00 | 0.85 | 0.01 | 0.54 | 0.01 | 0.85 | 0.02 | 0.15 |
| Burst Usage | 0.38 | 0.99 | 0.60 | 0.92 | 0.41 | 1.00 | 0.59 | 0.61 |
| Persistent Usage | 0.16 | 0.63 | 0.15 | 0.46 | 0.15 | 0.59 | 0.16 | 0.29 |
| Multi-provider Usage | 0.13 | 0.33 | 0.18 | 0.20 | 0.13 | 0.32 | 0.19 | 0.13 |
| Low Inpatient Rate | 0.11 | 0.92 | 0.91 | 0.28 | 0.03 | 0.81 | 0.92 | 0.26 |
| Low Acuity | 0.07 | 0.82 | 0.48 | 0.20 | 0.06 | 0.72 | 0.48 | 0.17 |
| FU Outpatient | NA | NA | NA | NA | NA | NA | NA | NA |
| FU NHS 111 Calls | 0.20 | 0.47 | 0.13 | 0.50 | 0.12 | 0.46 | 0.14 | 0.62 |
| FU Ambulance | 0.50 | 0.50 | 0.06 | 0.84 | 0.45 | 0.56 | 0.07 | 1.00 |
| Mental Health Medications | NA | NA | NA | NA | NA | NA | NA | NA |
| Neurological Medications | NA | NA | NA | NA | NA | NA | NA | NA |
| Hyp/ HF Medications | NA | NA | NA | NA | NA | NA | NA | NA |
| CHD/ Vascular Medications | NA | NA | NA | NA | NA | NA | NA | NA |
| Diabetes Medications | NA | NA | NA | NA | NA | NA | NA | NA |
| Respiratory Medications | NA | NA | NA | NA | NA | NA | NA | NA |
| Physical Comorbidities (1+) | 0.32 | 0.29 | 0.23 | 0.32 | 0.34 | 0.31 | 0.25 | 0.28 |

*Table reports class-conditional response probabilities for each indicator variable. Only probabilities of a “Yes” response are shown (i.e., P[Yes] given class membership).*

*Hyp = Hypertension; HF = Heart Failure, CHD = Coronary Heart Disease.*

# Appendix 7: Three Class Solution Tables

**Table A7.1: Demographic Variables by Latent Class Membership, N (%)**

|  | **HES** | | | **CURED** | | |
| --- | --- | --- | --- | --- | --- | --- |
|  | **Class 1,**  **N = 23,034,**  **(43.2%)** | **Class 2,**  **N =6,288,**  **(11.8%)** | **Class 3,**  **N = 24,028,**  **(45.0%)** | **Class 1,**  **N = 7,081,**  **(32.7%)** | **Class 2,**  **N =3,456,**  **(15.9%)** | **Class 3,**  **N = 11,139,**  **(51.4%)** |
| **Age** |  |  |  |  |  |  |
| 0 to 19 | 125 (0.5) | 192 (3.1) | 1,614 (6.7) | 65 (0.9) | 154 (4.5) | 726 (6.5) |
| 20 to 29 | 832 (3.6) | 1,287 (20.5) | 7,833 (32.7) | 401 (5.7) | 729 (21.1) | 2,973 (26.7) |
| 0 to 39 | 1,160 (5.0) | 1,142 (18.2) | 5,522 (23.1) | 436 (6.2) | 565 (16.3) | 2,078 (18.7) |
| 40 to 49 | 1,813 (7.9) | 1,061 (16.9) | 3,508 (14.7) | 521 (7.4) | 610 (17.7) | 1,693 (15.2) |
| 50 to 59 | 2,702 (11.7) | 918 (14.6) | 2,455 (10.3) | 628 (8.9) | 432 (12.5) | 1,136 (10.2) |
| 60 to 69 | 3,448 (15.0) | 605 (9.6) | 1,153 (4.8) | 881 (12.4) | 307 (8.9) | 794 (7.1) |
| 70 to 79 | 5,247 (22.8) | 524 (8.3) | 856 (3.6) | 1,568 (22.1) | 314 (9.1) | 782 (7.0) |
| 80+ | 7,682 (33.4) | 553 (8.8) | 990 (4.1) | 2,581 (36.4) | 345 (10.0) | 957 (8.6) |
| Missing | 25 | 6 | 97 | 0 | 0 | 0 |
| **Sex** |  |  |  |  |  |  |
| Female | 11,928 (52.0) | 3,434 (55.1) | 12,605 (53.5) | 3,830 (54.1) | 1,680 (48.6) | 5,650 (50.7) |
| Male | 10,993 (48.0) | 2,797 (44.9) | 10,969 (46.5) | 3,251 (45.9) | 1,776 (51.4) | 5,488 (49.3) |
| Missing | 113 | 57 | 454 | 0 | 0 | 1 |
| **IMD Quintile** |  |  |  |  |  |  |
| 1 (most deprived) | 6,801 (29.5) | 2,562 (40.7) | 8,919 (37.2) | 3,215 (45.4) | 1,979 (57.3) | 5,759 (51.8) |
| 2 | 4,895 (21.3) | 1,434 (22.8) | 5,485 (22.9) | 1,366 (19.3) | 662 (19.2) | 2,026 (18.2) |
| 3 | 4,276 (18.6) | 997 (15.9) | 3,918 (16.3) | 1,088 (15.4) | 370 (10.7) | 1,496 (13.5) |
| 4 | 3,946 (17.1) | 739 (11.8) | 3,298 (13.8) | 872 (12.3) | 292 (8.5) | 1,169 (10.5) |
| 5 (least deprived) | 3,114 (13.5) | 556 (8.8) | 2,357 (9.8) | 538 (7.6) | 149 (4.3) | 665 (6) |
| Missing | 2 | 0 | 51 | 2 | 4 | 24 |
| **Ethnicity** |  |  |  |  |  |  |
| Asian | 1,431 (6.2) | 281 (4.5) | 1,559 (6.5) | 380 (5.4) | 204 (5.9) | 1,066 (9.6) |
| Black | 770 (3.3) | 258 (4.1) | 1,307 (5.4) | 47 (0.7) | 22 (0.6) | 152 (1.4) |
| Mixed | 133 (0.6) | 63 (1.0) | 380 (1.6) | 23 (0.3) | 24 (0.7) | 100 (0.9) |
| Other | 451 (2) | 127 (2.0) | 887 (3.7) | 43 (0.6) | 36 (1) | 186 (1.7) |
| Unknown | 1,652 (7.2) | 443 (7.0) | 2,946 (12.3) | 140 (2) | 79 (2.3) | 383 (3.4) |
| White | 18,597 (80.7) | 5,116 (81.4) | 16,949 (70.5) | 6,448 (91.1) | 3,091 (89.4) | 9,252 (83.1) |
| Missing | 0 | 0 | 0 | 0 | 0 | 0 |

**Table A7.2: ED Attendance Reasons by Latent Class Membership, N (%)**

|  | **HES** | | | **CUREd** | | |
| --- | --- | --- | --- | --- | --- | --- |
| **Attendance Reason ^1^** | **Class 1,**  **N = 23,034,**  **(43.2%)** | **Class 2,**  **N =6,288,**  **(11.8%)** | **Class 3,**  **N = 24,028,**  **(45.0%)** | **Class 1,**  **N = 7,081,**  **(32.7%)** | **Class 2,**  **N =3,456,**  **(15.9%)** | **Class 3,**  **N = 11,139,**  **(51.4%)** |
| CNS | 481 (2.3) | 225 (3.7) | 500 (2.3) | 101 (2.0) | 52 (1.8) | 122 (1.4) |
| Cardiac | 1,624 (7.7) | 225 (3.7) | 253 (1.1) | 206 (4.1) | 57 (1.9) | 85 (1.0) |
| Ear, Nose, Throat | 261 (1.2) | 34 (0.6) | 438 (2.0) | 44 (0.9) | 14 (0.5) | 133 (1.5) |
| Endocrine | 483 (2.3) | 120 (2.0) | 86 (0.4) | 35 (0.7) | 10 (0.3) | 9 (0.1) |
| Gastrointestinal | 1,917 (9) | 553 (9.2) | 1,946 (8.8) | 230 (4.6) | 116 (3.9) | 240 (2.7) |
| Genitourinary | 2,508 (11.8) | 514 (8.5) | 2,252 (10.1) | 318 (6.3) | 142 (4.8) | 563 (6.4) |
| Infection | 1,393 (6.6) | 148 (2.5) | 890 (4.0) | 293 (5.8) | 97 (3.3) | 527 (6.0) |
| Injury | 3,468 (16.4) | 1,608 (26.7) | 6,577 (29.6) | 455 (9.0) | 272 (9.2) | 1,354 (15.4) |
| Psychosocial problems | 701 (3.3) | 420 (7.0) | 1,131 (5.1) | 76 (1.5) | 114 (3.8) | 259 (3.0) |
| No classification | 2,606 (12.3) | 839 (13.9) | 3,475 (15.7) | 2,476 (49.0) | 1,749 (58.9) | 4,483 (51.1) |
| Nothing abnormal | 1,445 (6.8) | 523 (8.7) | 1,678 (7.6) | 87 (1.7) | 68 (2.3) | 243 (2.8) |
| Other | 663 (3.1) | 155 (2.6) | 1,453 (6.5) | 272 (5.4) | 182 (6.1) | 491 (5.6) |
| Respiratory | 2,523 (11.9) | 497 (8.3) | 1,005 (4.5) | 306 (6.1) | 78 (2.6) | 138 (1.6) |
| Vascular or haematological | 1,131 (5.3) | 162 (2.7) | 509 (2.3) | 151 (3.0) | 20 (0.7) | 126 (1.4) |
| Missing | 1,830 | 265 | 1,835 | 2,031 | 485 | 2,366 |

*1: Category definitions are provided in Appendix 1*

**Table A7.3: ED Investigations by Latent Class Membership, N (%)**

|  | **HES** | | | **CUREd** | | |
| --- | --- | --- | --- | --- | --- | --- |
| **Investigation ^1^** | **Class 1,**  **N = 23,034,**  **(43.2%)** | **Class 2,**  **N =6,288,**  **(11.8%)** | **Class 3,**  **N = 24,028,**  **(45.0%)** | **Class 1,**  **N = 7,081,**  **(32.7%)** | **Class 2,**  **N =3,456,**  **(15.9%)** | **Class 3,**  **N = 11,139,**  **(51.4%)** |
| Administrative/ Unknown | 2,076 (9.1) | 1,131 (18.1) | 6,012 (25.6) | 454 (6.4) | 1,477 (42.7) | 5,451 (49.0) |
| Blood Gas Analysis | 658 (2.9) | 154 (2.5) | 527 (2.2) | 20 (0.3) | 5 (0.1) | 6 (0.1) |
| Biochemistry | 4,980 (21.8) | 1,434 (22.9) | 3,812 (16.2) | 1,237 (17.5) | 388 (11.2) | 933 (8.4) |
| Blood Typing | 33 (0.1) | 4 (0.1) | 48 (0.2) | 5 (0.1) | 0 (0.0) | 3 (0.0) |
| Cardiac Markers | 41 (0.2) | 4 (0.1) | 32 (0.1) | 7 (0.1) | 0 (0.0) | 3 (0.0) |
| Cardiac Tests | 2,157 (9.4) | 493 (7.9) | 1,366 (5.8) | 771 (10.9) | 239 (6.9) | 521 (4.7) |
| Haematology | 4,032 (17.6) | 1,156 (18.5) | 3,081 (13.1) | 1,087 (15.4) | 313 (9.1) | 608 (5.5) |
| Immunology | 7 (0.0) | 0 (0.0) | 30 (0.1) | 6 (0.1) | 0 (0.0) | 3 (0.0) |
| Microbiology | 214 (0.9) | 29 (0.5) | 201 (0.9) | 11 (0.2) | 1 (0.0) | 8 (0.1) |
| Other (no mapping) | 0 (0.0) | 0 (0.0) | 2 (0.0) | 0 (0.0) | 0 (0.0) | 0 (0.0) |
| Ophthalmology/Dental | 239 (1) | 48 (0.8) | 634 (2.7) | 0 (0.0) | 0 (0.0) | 0 (0.0) |
| Other/ Unspecified | 294 (1.3) | 100 (1.6) | 692 (2.9) | 223 (3.2) | 56 (1.6) | 140 (1.3) |
| Pregnancy Tests | 11 (0.0) | 6 (0.1) | 135 (0.6) | 2 (0.0) | 3 (0.1) | 25 (0.2) |
| Radiology/ Imaging | 7,386 (32.3) | 1,520 (24.3) | 5,823 (24.8) | 3,113 (44) | 908 (26.3) | 3,186 (28.6) |
| Toxicology | 12 (0.1) | 9 (0.1) | 33 (0.1) | 0 (0.0) | 0 (0.0) | 0 (0.0) |
| Urinalysis | 737 (3.2) | 170 (2.7) | 1,041 (4.4) | 143 (2) | 66 (1.9) | 238 (2.1) |
| Missing | 157 | 30 | 559 | 2 | 0 | 14 |

*1: Category definitions are provided in Appendix 2*

**Table A7.4: ED Treatments by Latent Class Membership, N (%)**

|  | **HES** | | | **CUREd** | | |
| --- | --- | --- | --- | --- | --- | --- |
| **ED Treatment ^1^** | **Class 1,**  **N = 23,034,**  **(43.2%)** | **Class 2,**  **N =6,288,**  **(11.8%)** | **Class 3,**  **N = 24,028,**  **(45.0%)** | **Class 1,**  **N = 7,081,**  **(32.7%)** | **Class 2,**  **N =3,456,**  **(15.9%)** | **Class 3,**  **N = 11,139,**  **(51.4%)** |
| Airway & respiratory support | 124 (0.5) | 12 (0.2) | 36 (0.2) | 761 (10.8) | 332 (9.6) | 663 (6.0) |
| Critical cardiovascular care | 18 (0.1) | 2 (0.0) | 7 (0.0) | 51 (0.7) | 14 (0.4) | 17 (0.2) |
| Deprecated code | 0 (0.0) | 0 (0.0) | 3 (0.0) | 0 (0.0) | 0 (0.0) | 0 (0.0) |
| Decontamination/poisoning management | 4 (0.0) | 1 (0.0) | 15 (0.1) | 0 (0.0) | 0 (0.0) | 3 (0.0) |
| Fracture and musculoskeletal care | 342 (1.5) | 78 (1.2) | 556 (2.3) | 8 (0.1) | 11 (0.3) | 108 (1.0) |
| Guidance | 4,620 (20.2) | 1,547 (24.6) | 7,885 (33.0) | 1,363 (19.3) | 1,090 (31.5) | 4,766 (42.8) |
| Invasive lines & fluid management | 3,871 (16.9) | 747 (11.9) | 1,697 (7.1) | 931 (13.2) | 187 (5.4) | 329 (3.0) |
| Medication & pharmacological | 4,368 (19.1) | 1,052 (16.8) | 2,868 (12.0) | 1,150 (16.3) | 408 (11.8) | 879 (7.9) |
| Minor surgical interventions | 270 (1.2) | 54 (0.9) | 659 (2.8) | 12 (0.2) | 10 (0.3) | 205 (1.8) |
| None | 1,305 (5.7) | 445 (7.1) | 2,271 (9.5) | 145 (2.1) | 215 (6.2) | 960 (8.6) |
| Observation, monitoring, review | 5,962 (26.1) | 1,734 (27.6) | 5,262 (22.0) | 1,170 (16.5) | 479 (13.9) | 1,240 (11.1) |
| Other | 1,469 (6.4) | 448 (7.1) | 1,518 (6.4) | 1,438 (20.3) | 675 (19.5) | 1,689 (15.2) |
| Pain and sedation | 24 (0.1) | 11 (0.2) | 50 (0.2) | 11 (0.2) | 4 (0.1) | 23 (0.2) |
| Social work | 8 (0.0) | 4 (0.1) | 18 (0.1) | 2 (0.0) | 0 (0.0) | 0 (0.0) |
| Wound management | 500 (2.2) | 145 (2.3) | 1,033 (4.3) | 30 (0.4) | 30 (0.9) | 248 (2.2) |
| Missing | 149 | 8 | 150 | 9 | 1 | 9 |

*1: Category definitions are provided in Appendix 2*

**Table A7.5: ED Arrival Mode and Discharge Destination by Latent Class Membership, N (%)**

|  | **HES** | | | **CUREd** | | |
| --- | --- | --- | --- | --- | --- | --- |
|  | **Class 1,**  **N = 23,034,**  **(43.2%)** | **Class 2,**  **N =6,288,**  **(11.8%)** | **Class 3,**  **N = 24,028,**  **(45.0%)** | **Class 1,**  **N = 7,081,**  **(32.7%)** | **Class 2,**  **N =3,456,**  **(15.9%)** | **Class 3,**  **N = 11,139,**  **(51.4%)** |
| **Arrival Mode ^1^** |  |  |  |  |  |  |
| Ambulance (including helicopter) | 13,423 (58.5) | 3,223 (51.4) | 5,349 (22.3) | 5,825 (82.3) | 2,275 (66.0) | 3,123 (28.3) |
| Other | 9,528 (41.5) | 3,052 (48.6) | 18,606 (77.7) | 1,252 (17.7) | 1,173 (34.0) | 7,903 (71.7) |
| Missing | 83 | 13 | 73 | 4 | 8 | 113 |
|  |  |  |  |  |  |  |
| **Discharge destination ^1^** |  |  |  |  |  |  |
| Discharged (home or elsewhere) | 10,097 (43.8) | 4,007 (63.7) | 16,998 (70.7) | 1,058 (14.9) | 1,933 (55.9) | 7,939 (71.4) |
| Referred to ED ward | 125 (0.5) | 37 (0.6) | 320 (1.3) | 6 (0.1) | 17 (0.5) | 439 (3.9) |
| Referred ambulatory/ community care | 32 (0.1) | 12 (0.2) | 82 (0.3) | 3 (0.0) | 7 (0.2) | 95 (0.9) |
| Referred to inpatient ward | 9,986 (43.4) | 1,394 (22.2) | 2,928 (12.2) | 5,951 (84.1) | 1,217 (35.2) | 1,528 (13.7) |
| Referred for procedure/outpatient | 193 (0.8) | 35 (0.6) | 437 (1.8) | 32 (0.5) | 26 (0.8) | 406 (3.6) |
| Transferred | 483 (2.1) | 107 (1.7) | 428 (1.8) | 7 (0.1) | 2 (0.1) | 50 (0.4) |
| Other | 231 (1.0) | 176 (2.8) | 803 (3.3) | 20 (0.3) | 253 (7.3) | 668 (6) |
| Unknown | 1,887 (8.2) | 520 (8.3) | 2,032 (8.5) | 0 (0.0) | 0 (0.0) | 0 (0.0) |
| Missing | 0 | 0 | 0 | 4 | 1 | 14 |

*1: Category definitions are provided in Appendix 2*

**Table A7.6: Healthcare Utilisation and Costs by Latent Class Membership, mean (SD)**

|  | **HES** | | | **CUREd** | | |
| --- | --- | --- | --- | --- | --- | --- |
|  | **Class 1,**  **N = 23,034,**  **(43.2%)** | **Class 2,**  **N =6,288,**  **(11.8%)** | **Class 3,**  **N = 24,028,**  **(45.0%)** | **Class 1,**  **N = 7,081,**  **(32.7%)** | **Class 2,**  **N =3,456,**  **(15.9%)** | **Class 3,**  **N = 11,139,**  **(51.4%)** |
| **Costs (GBP, 2021/22)** |  |  |  |  |  |  |
| Total costs ^1^ | £20,460 (£18,289) | £23,367 (£25,119) | £7,679 (£11,404) | £18,287 (£11,382) | £17,075 (£14,826) | £4,611 (£5,646) |
| ED costs | £1,963 (£782) | £4,367 (£3,454) | £1,560 (£968) | £2,135 (£673) | £4,169 (£2,771) | £1,503 (£507) |
| Inpatient costs ^2^ | £14,921 (16,795) | £15,273 (£22,614) | £4,648 (£10,215) | £14,405 (£11,102) | £9,812 (£12,551) | £2,605 (£5,318) |
| Outpatient costs | £2,609 (£3,006) | £2,854 (£3,731) | £1,233 (£2,015) | NA | NA | NA |
| Critical care costs | £199 (£897) | £255 (£1,065) | £63 (£465) | NA | NA | NA |
| Ambulance costs | NA | NA | NA | £1,711 (£1,114) | £2,917 (£3,340) | £474 (£638) |
| NHS 111 telephone costs | NA | NA | NA | £36 (£87) | £178 (£722) | £29 (£79) |
| Medication costs | £768 (£934) | £617 (£1013) | £175 (£521) | NA | NA | NA |
|  |  |  |  |  |  |  |
| **Healthcare Utilisation** |  |  |  |  |  |  |
| ED | 6.1 (1.5) | 15.9 (13.5) | 6.5 (3.7) | 6 (1.2) | 15 (10.9) | 5.9 (1.2) |
| Inpatient ^2^ | 5.4 (9.1) | 7.0 (9.3) | 2.1 (3.7) | 4.5 (1.7) | 4.6 (4.2) | 1.1 (1.2) |
| Outpatient | 14.1 (15.8) | 14.8 (17.4) | 6.7 (9.8) | NA | NA | NA |
| Critical care | 0.1 (0.5) | 0.1 (0.6) | 0.0 (0.2) | NA | NA | NA |
| Ambulance | NA | NA | NA | 4.6 (3.2) | 8.1 (9.9) | 1.3 (1.8) |
| NHS 111 telephone | NA | NA | NA | 3.1 (4.8) | 10.1 (25.8) | 2.1 (3.8) |

*1: Total costs in HES include expenditures related to ED, inpatient, outpatient, critical care, and prescribed medications. In CUREd, total costs reflect ED, inpatient, ambulance, and NHS 111 telephone service use.*

*2: Inpatient costs and utilisation refer to all inpatient admissions in HES, and to ED-linked inpatient admissions in CUREd.*

# Appendix 8: Four Class Solution Tables

**Table A8.1: Demographic Variables by Latent Class Membership, N (%)**

|  | **HES** | | | | **CUREd** | | | |
| --- | --- | --- | --- | --- | --- | --- | --- | --- |
|  | **Class 1,**  **N = 15,909,**  **(29.8%)** | **Class 2,**  **N =5,790,**  **(10.9%)** | **Class 3,**  **N = 16,078,**  **(30.1%)** | **Class 4,**  **N = 15,573,**  **(29.2%)** | **Class 1,**  **N = 6,704,**  **(30.9%)** | **Class 2,**  **N =2,191,**  **(10.1%)** | **Class 3,**  **N = 11,381,**  **(52.5%)** | **Class 4,**  **N = 1,400,**  **(6.5%)** |
| **Age** |  |  |  |  |  |  |  |  |
| 0 to 19 | 28 (0.2) | 213 (3.7) | 1,309 (8.2) | 381 (2.5) | 60 (0.9) | 131 (6.0) | 738 (6.5) | 16 (1.1) |
| 20 to 29 | 167 (1.1) | 1,271 (22.0) | 5,802 (36.2) | 2,712 (17.5) | 370 (5.5) | 574 (26.2) | 3,020 (26.5) | 139 (9.9) |
| 0 to 39 | 349 (2.2) | 1,098 (19.0) | 3,782 (23.6) | 2,595 (16.7) | 421 (6.3) | 424 (19.4) | 2,119 (18.6) | 115 (8.2) |
| 40 to 49 | 895 (5.6) | 976 (16.9) | 2,203 (13.8) | 2,308 (14.9) | 473 (7.1) | 430 (19.6) | 1,732 (15.2) | 189 (13.5) |
| 50 to 59 | 1,782 (11.2) | 833 (14.4) | 1,456 (9.1) | 2,004 (12.9) | 599 (8.9) | 277 (12.6) | 1,151 (10.1) | 169 (12.1) |
| 60 to 69 | 2,667 (16.8) | 521 (9.0) | 647 (4.0) | 1,371 (8.8) | 840 (12.5) | 159 (7.3) | 814 (7.2) | 169 (12.1) |
| 70 to 79 | 4,199 (26.4) | 426 (7.4) | 409 (2.6) | 1,593 (10.3) | 1,502 (22.4) | 99 (4.5) | 812 (7.1) | 251 (17.9) |
| 80+ | 5,807 (36.5) | 447 (7.7) | 406 (2.5) | 2,565 (16.5) | 2,439 (36.4) | 97 (4.4) | 995 (8.7) | 352 (25.1) |
| Missing | 15 | 5 | 64 | 44 | 0 | 0 | 0 | 0 |
| **Sex** |  |  |  |  |  |  |  |  |
| Female | 7,531 (47.6) | 3,124 (54.5) | 7,909 (50.4) | 9,403 (60.8) | 3,606 (53.8) | 1,038 (47.4) | 5,781 (50.8) | 735 (52.5) |
| Male | 8,302 (52.4) | 2,613 (45.5) | 7,771 (49.6) | 6,073 (39.2) | 3,098 (46.2) | 1,153 (52.6) | 5,599 (49.2) | 665 (47.5) |
| Missing | 76 | 53 | 398 | 97 | 0 | 0 | 1 | 0 |
| **IMD Quintile** |  |  |  |  |  |  |  |  |
| 1 (most deprived) | 4,757 (29.9) | 2,387 (41.2) | 6,149 (38.3) | 4,989 (32.1) | 3,044 (45.4) | 1,271 (58.1) | 5888 (51.8) | 750 (53.6) |
| 2 | 3,417 (21.5) | 1,298 (22.4) | 3,711 (23.1) | 3,388 (21.8) | 1,284 (19.2) | 433 (19.8) | 2073 (18.3) | 264 (18.9) |
| 3 | 2,942 (18.5) | 940 (16.2) | 2,580 (16.1) | 2,729 (17.5) | 1,033 (15.4) | 224 (10.2) | 1527 (13.4) | 170 (12.2) |
| 4 | 2,702 (17.0) | 660 (11.4) | 2,108 (13.1) | 2,513 (16.2) | 828 (12.4) | 175 (8.0) | 1189 (10.5) | 141 (10.1) |
| 5 (least deprived) | 2,090 (13.1) | 505 (8.7) | 1,498 (9.3) | 1,934 (12.4) | 513 (7.7) | 85 (3.9) | 680 (6) | 74 (5.3) |
| Missing | 1 | 0 | 32 | 20 | 2 | 3 | 24 | 1 |
| **Ethnicity** |  |  |  |  |  |  |  |  |
| Asian | 1,199 (7.5) | 258 (4.5) | 1,122 (7) | 692 (4.4) | 358 (5.3) | 132 (6.0) | 1,086 (9.5) | 74 (5.3) |
| Black | 534 (3.4) | 241 (4.2) | 982 (6.1) | 578 (3.7) | 44 (0.7) | 11 (0.5) | 154 (1.4) | 12 (0.9) |
| Mixed | 75 (0.5) | 67 (1.2) | 282 (1.8) | 152 (1) | 21 (0.3) | 17 (0.8) | 103 (0.9) | 6 (0.4) |
| Other | 332 (2.1) | 118 (2) | 677 (4.2) | 338 (2.2) | 41 (0.6) | 26 (1.2) | 191 (1.7) | 7 (0.5) |
| Unknown | 1,119 (7.0) | 416 (7.2) | 2,228 (13.9) | 1,278 (8.2) | 132 (2.0) | 64 (2.9) | 386 (3.4) | 20 (1.4) |
| White | 12,650 (79.5) | 4,690 (81.0) | 10,787 (67.1) | 12,535 (80.5) | 6,108 (91.1) | 1,941 (88.6) | 9,461 (83.1) | 1,281 (91.5) |
| Missing | 0 | 0 | 0 | 0 | 0 | 0 | 0 | 0 |

**Table A8.2: ED Attendance Reasons by Latent Class Membership, N (%)**

|  | **HES** | | | | **CUREd** | | | |
| --- | --- | --- | --- | --- | --- | --- | --- | --- |
| **Attendance Reason ^1^** | **Class 1,**  **N = 15,909,**  **(29.8%)** | **Class 2,**  **N =5,790,**  **(10.9%)** | **Class 3,**  **N = 16,078,**  **(30.1%)** | **Class 4,**  **N = 15,573,**  **(29.2%)** | **Class 1,**  **N = 6,704,**  **(30.9%)** | **Class 2,**  **N =2,191,**  **(10.1%)** | **Class 3,**  **N = 11,381,**  **(52.5%)** | **Class 4,**  **N = 1,400,**  **(6.5%)** |
| CNS | 272 (1.9) | 188 (3.4) | 195 (1.3) | 551 (3.8) | 95 (2.0) | 33 (1.7) | 125 (1.4) | 22 (2.0) |
| Cardiac | 1,400 (9.6) | 184 (3.3) | 148 (1.0) | 370 (2.6) | 198 (4.2) | 15 (0.8) | 86 (1.0) | 49 (4.5) |
| Ear, Nose, Throat | 193 (1.3) | 32 (0.6) | 352 (2.4) | 156 (1.1) | 40 (0.8) | 7 (0.4) | 134 (1.5) | 10 (0.9) |
| Endocrine | 323 (2.2) | 108 (1.9) | 29 (0.2) | 229 (1.6) | 31 (0.7) | 4 (0.2) | 10 (0.1) | 9 (0.8) |
| Gastrointestinal | 1,233 (8.4) | 494 (8.9) | 1,142 (7.7) | 1,547 (10.7) | 219 (4.6) | 70 (3.5) | 247 (2.8) | 50 (4.6) |
| Genitourinary | 1,719 (11.8) | 482 (8.7) | 1,446 (9.8) | 1,627 (11.3) | 296 (6.2) | 87 (4.4) | 574 (6.4) | 66 (6.1) |
| Infection | 984 (6.7) | 124 (2.2) | 567 (3.8) | 756 (5.2) | 282 (5.9) | 60 (3.0) | 538 (6.0) | 37 (3.4) |
| Injury | 2,214 (15.2) | 1,546 (27.8) | 4,699 (31.7) | 3,194 (22.1) | 434 (9.1) | 211 (10.7) | 1,372 (15.3) | 64 (5.9) |
| Psychosocial problems | 434 (3.0) | 423 (7.6) | 701 (4.7) | 694 (4.8) | 70 (1.5) | 84 (4.3) | 263 (2.9) | 32 (2.9) |
| No classification | 1,828 (12.5) | 798 (14.4) | 2,462 (16.6) | 1,832 (12.7) | 2,316 (48.7) | 1,198 (60.8) | 4,607 (51.3) | 587 (53.9) |
| Nothing abnormal | 979 (6.7) | 479 (8.6) | 1,111 (7.5) | 1,077 (7.5) | 78 (1.6) | 61 (3.1) | 249 (2.8) | 10 (0.9) |
| Other | 497 (3.4) | 137 (2.5) | 1,183 (8.0) | 454 (3.1) | 259 (5.4) | 110 (5.6) | 500 (5.6) | 76 (7.0) |
| Respiratory | 1,727 (11.8) | 413 (7.4) | 508 (3.4) | 1,377 (9.5) | 293 (6.2) | 24 (1.2) | 142 (1.6) | 63 (5.8) |
| Vascular or haematological | 805 (5.5) | 149 (2.7) | 270 (1.8) | 578 (4.0) | 143 (3.0) | 8 (0.4) | 132 (1.5) | 14 (1.3) |
| Missing | 1,301 | 233 | 1,265 | 1,131 | 1,950 | 219 | 2,402 | 311 |

*1: Category definitions are provided in Appendix 1*

**Table A8.3: ED Investigations by Latent Class Membership, N (%)**

|  | **HES** | | | | **CUREd** | | | |
| --- | --- | --- | --- | --- | --- | --- | --- | --- |
| **Investigation ^1^** | **Class 1,**  **N = 15,909,**  **(29.8%)** | **Class 2,**  **N =5,790,**  **(10.9%)** | **Class 3,**  **N = 16,078,**  **(30.1%)** | **Class 4,**  **N = 15,573,**  **(29.2%)** | **Class 1,**  **N = 6,704,**  **(30.9%)** | **Class 2,**  **N =2,191,**  **(10.1%)** | **Class 3,**  **N = 11,381,**  **(52.5%)** | **Class 4,**  **N = 1,400,**  **(6.5%)** |
| Administrative/ Unknown | 1,438 (9.1) | 1,089 (18.9) | 4,764 (30.5) | 1,928 (12.5) | 416 (6.2) | 1,237 (56.5) | 5,576 (49.1) | 153 (10.9) |
| Blood Gas Analysis | 428 (2.7) | 142 (2.5) | 284 (1.8) | 485 (3.1) | 20 (0.3) | 3 (0.1) | 6 (0.1) | 2 (0.1) |
| Biochemistry | 3,371 (21.3) | 1,288 (22.3) | 2,122 (13.6) | 3,445 (22.3) | 1,192 (17.8) | 203 (9.3) | 952 (8.4) | 211 (15.1) |
| Blood Typing | 26 (0.2) | 5 (0.1) | 32 (0.2) | 22 (0.1) | 5 (0.1) | 0 (0.0) | 3 (0.0) | 0 (0.0) |
| Cardiac Markers | 32 (0.2) | 5 (0.1) | 23 (0.1) | 17 (0.1) | 7 (0.1) | 0 (0.0) | 3 (0.0) | 0 (0.0) |
| Cardiac Tests | 1,521 (9.6) | 441 (7.6) | 774 (5.0) | 1,280 (8.3) | 724 (10.8) | 104 (4.7) | 539 (4.7) | 164 (11.7) |
| Haematology | 2,762 (17.5) | 1,081 (18.7) | 1,793 (11.5) | 2,633 (17.1) | 1,014 (15.1) | 137 (6.3) | 625 (5.5) | 232 (16.6) |
| Immunology | 5 (0.0) | 1 (0.0) | 21 (0.1) | 10 (0.1) | 6 (0.1) | 0 (0.0) | 3 (0.0) | 0 (0.0) |
| Microbiology | 146 (0.9) | 23 (0.4) | 140 (0.9) | 135 (0.9) | 11 (0.2) | 0 (0.0) | 8 (0.1) | 1 (0.1) |
| Other (no mapping) | 0 (0.0) | 0 (0.0) | 1 (0.0) | 1 (0.0) | 0 (0.0) | 0 (0.0) | 0 (0.0) | 0 (0.0) |
| Ophthalmology/Dental | 186 (1.2) | 47 (0.8) | 520 (3.3) | 168 (1.1) | 0 (0.0) | 0 (0.0) | 0 (0.0) | 0 (0.0) |
| Other/ Unspecified | 208 (1.3) | 94 (1.6) | 547 (3.5) | 237 (1.5) | 208 (3.1) | 23 (1.0) | 142 (1.2) | 46 (3.3) |
| Pregnancy Tests | 2 (0.0) | 6 (0.1) | 107 (0.7) | 37 (0.2) | 1 (0.0) | 1 (0.0) | 26 (0.2) | 2 (0.1) |
| Radiology/ Imaging | 5,200 (32.9) | 1,373 (23.8) | 3,759 (24.1) | 4,397 (28.5) | 2,961 (44.2) | 445 (20.3) | 3,243 (28.5) | 558 (39.9) |
| Toxicology | 5 (0.0) | 10 (0.2) | 19 (0.1) | 20 (0.1) | 0 (0.0) | 0 (0.0) | 0 (0.0) | 0 (0.0) |
| Urinalysis | 463 (2.9) | 167 (2.9) | 698 (4.5) | 620 (4.0) | 137 (2.0) | 38 (1.7) | 241 (2.1) | 31 (2.2) |
| Missing | 116 | 18 | 474 | 138 | 2 | 0 | 14 | 0 |

*1: Category definitions are provided in Appendix 2*

**Table A8.4: ED Treatments by Latent Class Membership, N (%)**

|  | **HES** | | | | **CUREd** | | | |
| --- | --- | --- | --- | --- | --- | --- | --- | --- |
| **ED Treatment ^1^** | **Class 1,**  **N = 15,909,**  **(29.8%)** | **Class 2,**  **N =5,790,**  **(10.9%)** | **Class 3,**  **N = 16,078,**  **(30.1%)** | **Class 4,**  **N = 15,573,**  **(29.2%)** | **Class 1,**  **N = 6,704,**  **(30.9%)** | **Class 2,**  **N =2,191,**  **(10.1%)** | **Class 3,**  **N = 11,381,**  **(52.5%)** | **Class 4,**  **N = 1,400,**  **(6.5%)** |
| Airway & respiratory support | 80 (0.5) | 10 (0.2) | 11 (0.1) | 71 (0.5) | 714 (10.7) | 167 (7.6) | 680 (6.0) | 195 (13.9) |
| Critical cardiovascular care | 14 (0.1) | 2 (0.0) | 3 (0.0) | 8 (0.1) | 49 (0.7) | 5 (0.2) | 17 (0.1) | 11 (0.8) |
| Deprecated code | 0 (0.0) | 0 (0.0) | 2 (0.0) | 1 (0.0) | 0 (0.0) | 0 (0.0) | 0 (0.0) | 0 (0.0) |
| Decontamination/poisoning management | 3 (0.0) | 0 (0.0) | 12 (0.1) | 5 (0.0) | 0 (0.0) | 0 (0.0) | 3 (0.0) | 0 (0.0) |
| Fracture and musculoskeletal care | 223 (1.4) | 73 (1.3) | 387 (2.4) | 293 (1.9) | 8 (0.1) | 7 (0.3) | 111 (1.0) | 1 (0.1) |
| Guidance | 3,189 (20.2) | 1,475 (25.5) | 5,792 (36.3) | 3,596 (23.2) | 1,276 (19.1) | 842 (38.4) | 4,859 (42.7) | 242 (17.3) |
| Invasive lines & fluid management | 2,676 (16.9) | 651 (11.3) | 803 (5.0) | 2,185 (14.1) | 902 (13.5) | 61 (2.8) | 336 (3.0) | 148 (10.6) |
| Medication & pharmacological | 2,931 (18.5) | 948 (16.4) | 1,588 (9.9) | 2,821 (18.2) | 1,081 (16.1) | 167 (7.6) | 901 (7.9) | 288 (20.6) |
| Minor surgical interventions | 186 (1.2) | 45 (0.8) | 482 (3.0) | 270 (1.7) | 12 (0.2) | 7 (0.3) | 206 (1.8) | 2 (0.1) |
| None | 889 (5.6) | 421 (7.3) | 1,719 (10.8) | 992 (6.4) | 140 (2.1) | 169 (7.7) | 981 (8.6) | 30 (2.1) |
| Observation, monitoring, review | 4,114 (26.0) | 1,592 (27.5) | 3,208 (20.1) | 4,044 (26.1) | 1,121 (16.7) | 301 (13.7) | 1,264 (11.1) | 203 (14.5) |
| Other | 1,095 (6.9) | 414 (7.2) | 1,059 (6.6) | 867 (5.6) | 1,354 (20.2) | 442 (20.2) | 1,735 (15.3) | 271 (19.4) |
| Pain and sedation | 17 (0.1) | 10 (0.2) | 42 (0.3) | 16 (0.1) | 11 (0.2) | 2 (0.1) | 23 (0.2) | 2 (0.1) |
| Social work | 3 (0.0) | 5 (0.1) | 15 (0.1) | 7 (0.0) | 2 (0.0) | 0 (0.0) | 0 (0.0) | 0 (0.0) |
| Wound management | 390 (2.5) | 138 (2.4) | 841 (5.3) | 309 (2.0) | 26 (0.4) | 21 (1.0) | 255 (2.2) | 6 (0.4) |
| Missing | 99 | 6 | 114 | 88 | 8 | 0 | 10 | 1 |

*1: Category definitions are provided in Appendix 2*

**Table A8.5: ED Arrival Mode and Discharge Destination by Latent Class Membership, N (%)**

|  | **HES** | | | | **CUREd** | | | |
| --- | --- | --- | --- | --- | --- | --- | --- | --- |
|  | **Class 1,**  **N = 15,909,**  **(29.8%)** | **Class 2,**  **N =5,790,**  **(10.9%)** | **Class 3,**  **N = 16,078,**  **(30.1%)** | **Class 4,**  **N = 15,573,**  **(29.2%)** | **Class 1,**  **N = 6,704,**  **(30.9%)** | **Class 2,**  **N =2,191,**  **(10.1%)** | **Class 3,**  **N = 11,381,**  **(52.5%)** | **Class 4,**  **N = 1,400,**  **(6.5%)** |
| **Arrival Mode ^1^** |  |  |  |  |  |  |  |  |
| Ambulance (including helicopter) | 9,590 (60.5) | 2,897 (50.1) | 2,517 (15.7) | 6,991 (45.1) | 5,475 (81.7) | 1,227 (56.2) | 3,264 (29.0) | 1,257 (89.8) |
| Other | 6,274 (39.5) | 2,884 (49.9) | 13,516 (84.3) | 8,512 (54.9) | 1,225 (18.3) | 958 (43.8) | 8,002 (71.0) | 143 (10.2) |
| Missing | 45 | 9 | 45 | 70 | 4 | 6 | 115 | 0 |
|  |  |  |  |  |  |  |  |  |
| **Discharge destination ^1^** |  |  |  |  |  |  |  |  |
| Discharged (home or elsewhere) | 6,924 (43.5) | 3,799 (65.6) | 12,112 (75.3) | 8,267 (53.1) | 924 (13.8) | 1,562 (71.3) | 8,099 (71.3) | 345 (24.7) |
| Referred to ED ward | 104 (0.7) | 27 (0.5) | 282 (1.8) | 69 (0.4) | 6 (0.1) | 5 (0.2) | 451 (4.0) | 0 (0.0) |
| Referred ambulatory/ community care | 19 (0.1) | 11 (0.2) | 57 (0.4) | 39 (0.3) | 2 (0.0) | 5 (0.2) | 97 (0.9) | 1 (0.1) |
| Referred to inpatient ward | 6,987 (43.9) | 1,181 (20.4) | 979 (6.1) | 5,161 (33.1) | 5,717 (85.3) | 379 (17.3) | 1,577 (13.9) | 1,023 (73.2) |
| Referred for procedure/outpatient | 133 (0.8) | 26 (0.4) | 334 (2.1) | 172 (1.1) | 28 (0.4) | 19 (0.9) | 409 (3.6) | 8 (0.6) |
| Transferred | 299 (1.9) | 98 (1.7) | 266 (1.7) | 355 (2.3) | 7 (0.1) | 2 (0.1) | 50 (0.4) | 0 (0.0) |
| Other | 146 (0.9) | 170 (2.9) | 640 (4.0) | 254 (1.6) | 18 (0.3) | 219 (10.0) | 683 (6.0) | 21 (1.5) |
| Unknown | 1,297 (8.2) | 478 (8.3) | 1,408 (8.8) | 1,256 (8.1) | 0 (0.0) | 0 (0.0) | 0 (0.0) | 0 (0.0) |
| Missing | 0 | 0 | 0 | 0 | 2 | 0 | 15 | 2 |

*1: Category definitions are provided in Appendix 2*

**Table A8.6: Healthcare Utilisation and Costs by Latent Class Membership, mean (SD)**

|  | **HES** | | | | **CUREd** | | | |
| --- | --- | --- | --- | --- | --- | --- | --- | --- |
|  | **Class 1,**  **N = 15,909,**  **(29.8%)** | **Class 2,**  **N =5,790,**  **(10.9%)** | **Class 3,**  **N = 16,078,**  **(30.1%)** | **Class 4,**  **N = 15,573,**  **(29.2%)** | **Class 1,**  **N = 6,704,**  **(30.9%)** | **Class 2,**  **N =2,191,**  **(10.1%)** | **Class 3,**  **N = 11,381,**  **(52.5%)** | **Class 4,**  **N = 1,400,**  **(6.5%)** |
| **Costs (GBP, 2021/22)** |  |  |  |  |  |  |  |  |
| Total costs ^1^ | £21,097 (£18,782) | £23,030 (£25,525) | £4,927 (£8,678) | £16,297 (£16,046) | £18,368 (£11,347) | £13,277 (£11,753) | £4,704 (£5,736) | £24,352 (£16,348) |
| ED costs | £2,026 (£888) | £4,557 (£3,513) | £1,454 (£1,028) | £1,808 (£682) | £2,118 (£648) | £4,275 (£3,018) | £1,519 (£535) | £3,861 (£2,205) |
| Inpatient costs ^2^ | £15,443 (£17,218) | £14,911 (£23,005) | £2,573 (£8,050) | £11,431 (£14,534) | £14,560 (£11,067) | £6,072 (£8,735) | £2,656 (£5,383) | £16,995 (£14,766) |
| Outpatient costs | £2,628 (£3,065) | £2,743 (£3,656) | £832 (£1,424) | £2,350 (£2,864) | NA | NA | NA | NA |
| Critical care costs | £202 (£910) | £250 (£1,067) | £28 (£294) | £168 (£786) | NA | NA | NA | NA |
| Ambulance costs | NA | NA | NA | NA | £1,658 (£1,092) | £2,713 (£3,773) | £498 (£690) | £3,397 (£2,140) |
| NHS 111 telephone costs | NA | NA | NA | NA | £32 (£81) | £217 (£834) | £31 (£158) | £98 (£209) |
| Medication costs | £799 (£867) | £569 (£966) | £86 (£282) | £539 (£965) | NA | NA | NA | NA |
|  |  |  |  |  |  |  |  |  |
| **Healthcare Utilisation** |  |  |  |  |  |  |  |  |
| ED | 6.3 (2.1) | 16.8 (13.7) | 6.6 (4.2) | 6.0 (1.2) | 6.0 (1.1) | 16.9 (12.8) | 6.0 (1.4) | 11.1 (5.3) |
| Inpatient ^2^ | 5.6 (10.1) | 6.9 (9.1) | 1.3 (1.9) | 4.5 (6.2) | 4.5 (1.6) | 3.3 (3.3) | 1.1 (1.2) | 6.8 (4.3) |
| Outpatient | 14.2 (16.4) | 14.2 (16.6) | 4.6 (7.2) | 12.6 (14.0) | NA | NA | NA | NA |
| Critical care | 0.1 (0.5) | 0.1 (0.6) | 0.0 (0.1) | 0.1 (0.4) | NA | NA | NA | NA |
| Ambulance | NA | NA | NA | NA | 4.5 (3.1) | 7.6 (11.3) | 1.4 (1.9) | 9.3 (6.1) |
| NHS 111 telephone | NA | NA | NA | NA | 2.8 (4.5) | 11.8 (30.1) | 2.2 (5.5) | 7.1 (9.8) |

*1: Total costs in HES include expenditures related to ED, inpatient, outpatient, critical care, and prescribed medications. In CUREd, total costs reflect ED, inpatient, ambulance, and NHS 111 telephone service use.*

*2: Inpatient costs and utilisation refer to all inpatient admissions in HES, and to ED-linked inpatient admissions in CUREd.*

# Appendix 9: Cross Study Comparison of LCAs in FU Populations

**Table A9.1**

| **Study** | **Population** | **Class Descriptions** |
| --- | --- | --- |
| General population Studies | | |
| Current study | 43,350 FUs of ED in England, UK (HES) | 1. Chronic, Complex, & Frail (30%) 2. High-Intensity (11%) 3. Lower Severity and Morbidity (30%) 4. Moderate Morbidity, Complex-Need (29%) |
| Current study | 21,676 FUs of EDs from Yorkshire & Humber, England, UK (CUREd) | 1. Chronic, Complex, & Frail (31%) 2. High-Intensity (10%) 3. Lower Severity and Morbidity (53%) 4. High intensity, high need (29%) |
| Chiu (2022) (7) | 13,676 adult FUs with chronic conditions, Quebec, Canada | 1. High physical comorbidity (23%) 2. Mental health or alcohol/substance abuse (17%) 3. Low morbidity (40%) 4. Injury or chronic non-cancer pain (20%) |
| Birmingham (2020) (6) | 5,731 FUs of EDs, a Midwestern hospital USA | 1. Heart related [i.e. physical comorbidities] (4%) 2. Long-term [with high mental health prevalence] (35%) 3. Minor case [with high rates of musculoskeletal diagnosis, and low rates of inpatient admissions] (20%) 4. Short-term [with low inpatient admission rate] (40%) |
| Specific population studies | | |
| Dufour (2021) (9) | 21,393 geriatric FUs, Quebec Canada | 1. People with pulmonary and cardiac disease (18%) 2. People with dementia and mental health disorders (10%) 3. Low comorbidity (39%) 4. People with cancer (33%) |
| Mitchell (2017) (10) | 1,988 Homeless FUs, Boston USA | 1. Older persons with chronic illness (10%) 2. Persons with mental illness and disability (28%) 3. Young healthy persons (6%) 4. Persons with alcohol use disorders (15%) 5. Young persons with drug use and co-occurring disorders (18%) 6. Persons with tri-morbid (drug and alcohol, mental health and health conditions) illness (24%) |

*Note the study by di Bella et al. (2020) (8) was excluded from this table as the sample included both non-FUs and FUs.*
